# History of the geographic distribution of the western blacklegged tick, *Ixodes pacificus*, in the United States

**DOI:** 10.1016/j.ttbdis.2024.102325

**Published:** 2024-02-21

**Authors:** Lars Eisen, Megan E.M. Saunders, Vicki L. Kramer, Rebecca J. Eisen

**Affiliations:** a Division of Vector-Borne Diseases, National Center for Emerging and Zoonotic Infectious Diseases, Centers for Disease Control and Prevention, 3156 Rampart Road, Fort Collins, CO 80521, United States; b Vector-Borne Disease Section, California Department of Public Health, 1616 Capitol Ave, Sacramento, CA 95814, United States

**Keywords:** *Ixodes pacificus*, Geographic distribution, United States

## Abstract

*Ixodes pacificus* (the western blacklegged tick) occurs in the far western United States (US), where it commonly bites humans. This tick was not considered a species of medical concern until it was implicated in the 1980s as a vector of Lyme disease spirochetes. Later, it was discovered to also be the primary vector to humans in the far western US of agents causing anaplasmosis and hard tick relapsing fever. The core distribution of *I. pacificus* in the US includes California, western Oregon, and western Washington, with outlier populations reported in Utah and Arizona. In this review, we provide a history of the documented occurrence of *I. pacificus* in the US from the 1890s to present, and discuss associations of its geographic range with landscape, hosts, and climate. In contrast to *Ixodes scapularis* (the blacklegged tick) in the eastern US, there is no evidence for a dramatic change in the geographic distribution of *I. pacificus* over the last half-century. Field surveys in the 1930s and 1940s documented *I. pacificus* along the Pacific Coast from southern California to northern Washington, in the Sierra Nevada foothills, and in western Utah. County level collection records often included both immatures and adults of *I. pacificus*, recovered by drag sampling or from humans, domestic animals, and wildlife. The estimated geographic distribution presented for *I. pacificus* in 1945 by Bishopp and Trembley is similar to that presented in 2022 by the Centers for Disease Control and Prevention. There is no clear evidence of range expansion for *I. pacificus*, separate from tick records in new areas that could have resulted from newly initiated or intensified surveillance efforts. Moreover, there is no evidence from long-term studies that the density of questing *I. pacificus* ticks has increased over time in specific areas. It therefore is not surprising that the incidence of Lyme disease has remained stable in the Pacific Coast states from the early 1990s, when it became a notifiable condition, to present. We note that deforestation and deer depredation were less severe in the far western US during the 1800s and early 1900s compared to the eastern US. This likely contributed to *I. pacificus* maintaining stable, widespread populations across its geographic range in the far western US in the early 1900s, while *I. scapularis* during the same time period appears to have been restricted to a small number of geographically isolated refugia sites within its present range in the eastern US. The impact that a warming climate may have had on the geographic distribution and local abundance of *I. pacificus* in recent decades remains unclear.

## 1. Introduction

**Fig. 1.**
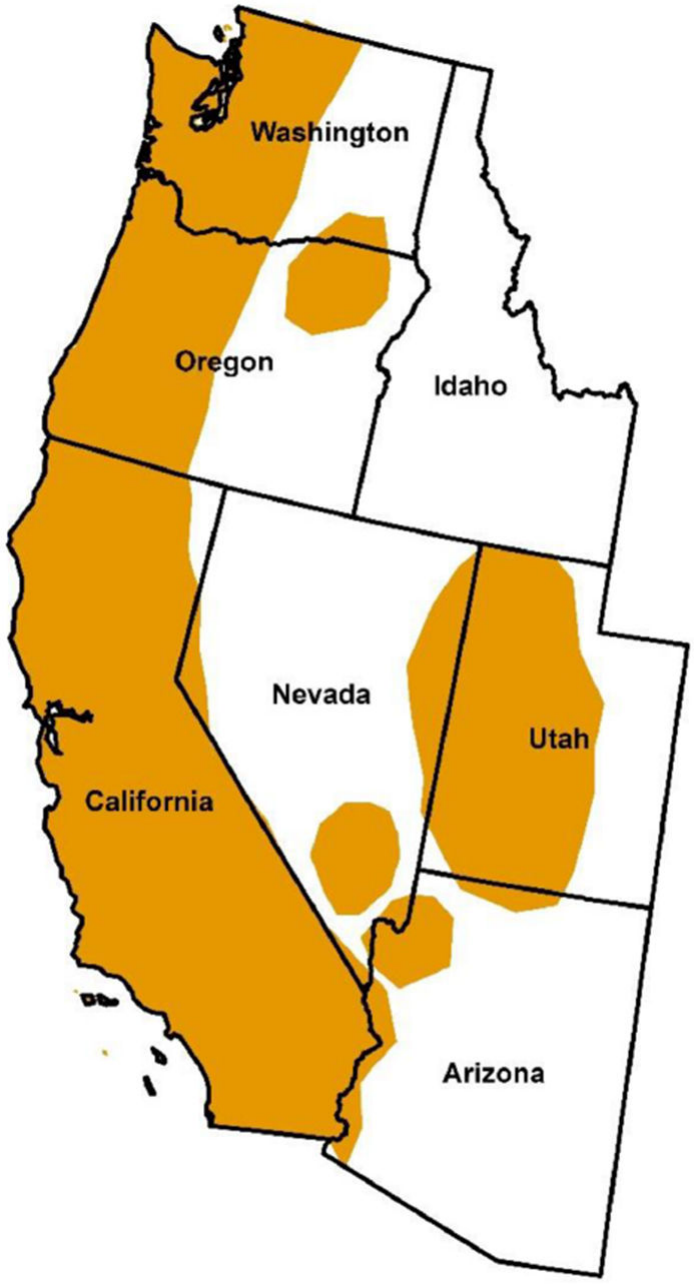
Estimated geographic distribution of *Ixodes pacificus* in the western United States (from CDC, 2023a).

*Ixodes pacificus* (the western blacklegged tick) occurs in far western North America, from Baja California in Mexico in the south through the United States (US) and into British Columbia in Canada in the north (Guzmán-Cornejo and Robbins, 2010; Eisen et al., 2016a; Lindquist et al., 2016). In the US, the core distribution includes California, western Oregon, and western Washington, with outlier records from Arizona and Utah (Fig. 1). Key hosts for the immature life stages of *I. pacificus* include lizards, rodents, and birds, whereas the adults predominantly parasitize large mammals (Furman and Loomis, 1984; Durden and Keirans, 1996; Castro and Wright, 2007; McVicar et al., 2022). The primary reproductive host for *I. pacificus* adults is *Odocoileus hemionus*, including the subspecies *Odocoileus hemionus hemionus*, mule deer, and *Odocoileus hemionus columbianus*, Columbian black-tailed deer (Heffelfinger and Latch, 2003). *Odocoileus hemionus columbianus* occurs in the Coast Ranges from northern California through Oregon and Washington, and *O. hemionus hemionus* is found in the remaining parts of the geographic range of *I. pacificus*. In this paper, both subspecies are referred to simply as deer. There also are geographically limited populations of a subspecies of the white-tailed deer (Columbian white-tailed deer, *Odocoileus virginianus leucurus*) present in western Oregon and Washington (Gavin, 1984; Fulbright, 2011). The role of other types of animals, such as mid- to large-sized carnivores, as alternative reproductive hosts for *I. pacificus* is not clear.

*Ixodes pacificus* has long been recognized to bite humans and domestic animals (Cooley and Kohls, 1945). However, it was not considered a species of medical concern until the recognition in the late 1970s and early 1980s that Lyme disease cases occurred in California (Steere and Malawista, 1979; Lane and Lavoie, 1988), and *I. pacificus* served as a vector of Lyme disease spirochetes (Burgdorfer et al., 1985). Similar to *Ixodes scapularis* (the blacklegged tick) in the eastern US, *I. pacificus* has emerged as the preeminent tick vector of human pathogens in the far western US in the last half-century. More than 4000 *I. pacificus* ticks have been recorded to infest humans in the US (Eisen, 2022), and this tick species is considered the primary vector in the far west of three bacterial agents known to cause human disease: *Anaplasma phagocytophilum* causing anaplasmosis, *Borrelia burgdorferi* sensu stricto (s.s.) causing Lyme disease, and *Borrelia miyamotoi* causing hard tick relapsing fever (Eisen and Paddock, 2021). *Ixodes pacificus* also is naturally infected with other species within the *Borrelia burgdorferi* sensu lato (s.l.) complex with unclear pathogenicity to humans, including *Borrelia americana, Borrelia bissettiae, Borrelia californiensis*, and *Borrelia lanei* (Rudenko et al., 2011; Fedorova et al., 2014; Margos et al., 2017; Rose et al., 2019; Xu et al., 2019; MacDonald et al., 2020a; Salkeld et al., 2021; Osikowicz et al., 2024).

In this review, we provide a history of the documented occurrence of *I. pacificus* in the US, and discuss associations of its geographic range with landscape, hosts, and climate. Sources for *I. pacificus* records included published literature together with unpublished information from: (i) the United States National Tick Collection (USNTC), housed at Georgia Southern University, Statesboro, GA, USA (USNTC, 2023); and (ii) a database for tick records maintained by the California Department of Public Health (CDPH). For general reviews on the biology and ecology of *I. pacificus*, we refer to Arthur and Snow (1968), Lane et al. (1991), Castro and Wright (2007), Salkeld and Lane (2010), Eisen et al. (2016b), and McVicar et al. (2022).

## 2. Origin and population genetics of *I. pacificus*

*Ixodes pacificus* is a member of the *Ixodes ricinus* species complex, which includes the four primary *Ixodes* vectors of human disease agents in the northern hemisphere: *I. ricinus* (the castor bean tick) in Europe; *Ixodes persulcatus* (the taiga tick) in eastern Europe and Asia; *I. pacificus* in western North America; and *I. scapularis* in eastern North America (Keirans et al., 1999). Molecular phylogenetic analyses of this species complex indicate that *I. pacificus* is more closely related to *I. persulcatus* than to *I. scapularis* (Fukunaga et al., 2000; Xu et al., 2003). However, the evolutionary history of *I. pacificus* is poorly understood and it remains unclear when it arose as a species in far western North America and how it spread to achieve the current geographic distribution. Population genetic studies of *I. pacificus* have been very limited (Kain et al., 1997, 1999) and unable to clarify the processes that shaped the current geographic distribution of the tick. Kain et al. (1999) found a lack of discernable geographical patterns of differentiation in haplotypes for the mitochondrial DNA cytochrome oxidase III gene of *I. pacificus* within the core distributional area along the Pacific Coast (California, Oregon, and Washington in the US and British Columbia in Canada), whereas an outlier population in Utah was distinguishable from the populations within the core distributional area and also showed reduced haplotype diversity. The authors suggested that this ecologically isolated population, which is separated from the core distributional area of *I. pacificus* by a large expanse of arid high desert unsuitable for the tick, could have resulted from either range fragmentation during a Pleistocene glaciation event or a more recent introduction via host animals. We encourage renewed studies of the evolutionary history and recent geographic spread patterns of *I. pacificus* using state-of-the-science genetic molecular approaches.

## 3. History of the documented geographic distribution of *I. pacificus* in the US

Collection records of *I. pacificus* span 1893 to present. Cooley and Kohls (1943) described *I. pacificus* as a new species, but there are older records for the tick under other names: *Ixodes ricinus* (Neumann, 1896; Nuttall et al., 1911; Hearle, 1938), *Ixodes californicus* (Banks, 1908; Hooker et al., 1912; Gregson, 1942), *Ixodes ricinus* var. *californicus* (Clarke, 1912, 1913), and *Ixodes ricinus californicus* (Boynton and Woods, 1933; Jellison, 1934; Gregson, 1935; Davis and Kohls, 1937; Kohls and Cooley, 1937; Parker et al., 1937). The most common sources for records of the adult stage of *I. pacificus* are drag or flag sampling (from the 1930s onward) and hunter-killed deer. Early records of the immature stages involved specimens taken from hosts, especially lizards and rodents, whereas in recent decades immatures increasingly have been recovered by drag sampling in dense woodlands with a leaf litter ground cover and minimal emergent vegetation where larvae and nymphs are readily collected using this method. Moreover, in the Sierra Nevada foothills of California, *I. pacificus* nymphs were collected more frequently by flag sampling from logs, rocks, and tree trunks than from leaf litter (Hacker et al., 2021). Recently, there has been an increase in passive surveillance studies where *I. pacificus* ticks found infesting community members or their pets were submitted for identification by professional entomologists (Salkeld et al., 2019; Xu et al., 2019; Hahn et al., 2020; Porter et al., 2021). Benefits and drawbacks of such studies, including the issue of whether or not travel history is accounted for, was reviewed previously by Eisen and Eisen (2021).

### 3.1. Summary of the history of the documented geographic distribution in the US

In striking contrast to *I. scapularis* in the eastern US (see Eisen and Eisen, 2023), there has not been a dramatic change in the geographic distribution of *I. pacificus* over the last half-century (Eisen et al., 2016a). Collection records from 1893 to 1915 showed *I. pacificus* to be present in coastal and interior California and coastal Oregon, and extensive field surveys in the 1930s and 1940s documented the tick to occur along the Pacific Coast from southern California to northern Washington, as well as in the Sierra Nevada foothills of California and in western Utah (see Sections 3.2-3.5). By 1950, *I. pacificus* had been recorded from 34 counties in California, 12 counties in Oregon, seven counties in Washington, and three counties in Utah. Two maps depicting the geographic distribution of *I. pacificus* in the US were published in 1945 (Bishopp and Trembley, 1945; Cooley and Kohls, 1945). There was agreement that the tick occurred from southernmost California through Oregon to northernmost Washington: the continuous geographic range was estimated to include the western parts of these states but there also were scattered collection locations indicated in eastern California. The main difference between the maps was that Bishopp and Trembley also included outlier collection records from southwestern Utah. The estimated distribution for *I. pacificus* presented by Bishopp and Trembley in 1945 is very similar to the depiction of the tick’s distribution as of 2022 by the Centers for Disease Control and Prevention (CDC) (Fig. 1). The expanding documented geographic distribution of *I. pacificus* along the Sierra Nevada Range in eastern California since 1950 is likely related in large part to increased surveillance efforts in this part of the state following the recognition in the early 1980s of *I. pacificus* as a vector of *B. burgdorferi* s. s. and subsequent detection of local clusters of Lyme disease cases in the Sierra Nevada foothills (see Sections 3.2.2-3.2.3).

**Fig. 2.**
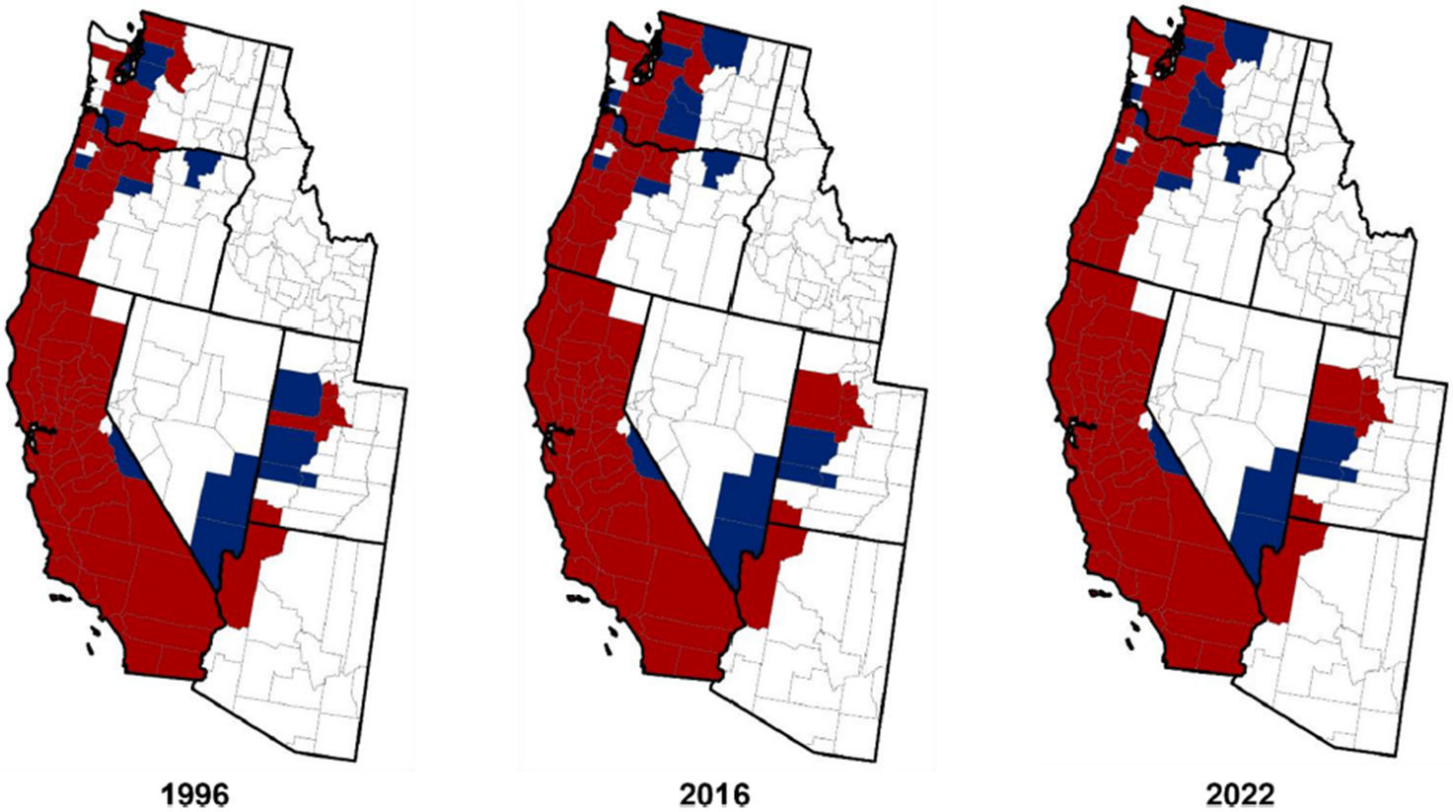
Distribution of counties in the western United States where *Ixodes pacificus* was classified as established (shown in red; 6 or more ticks of a single life stage or 2 or more life stages) or reported (shown in blue; fewer than six ticks and one life stage only) by 1996 (Dennis et al., 1998), 2015 (Eisen et al., 2016a), and 2022 (CDC, 2023c).

Collection records for *I. pacificus* accumulated steadily from California, Oregon, and Washington from the 1950s to the mid-1990s (see Sections 3.2.2, 3.3.2, and 3.4.2). Dennis et al. (1998) then presented the first summary of counties across the western US where *I. pacificus* had been reported or was considered established (i.e., at least six individuals of a single life stage or at least two life stages detected in the county), based on data up to 1996. Records came from literature review, a questionnaire sent to public health officials, acarologists, and Lyme disease researchers, and review of USNTC records. By 1996, *I. pacificus* was classified as established in 55 of 58 counties in California, nearly all counties in western Oregon, and most counties in western Washington (Fig. 2). Areas in these states where *I. pacificus* is absent or occurs only in isolated locations include high elevations in the Sierra Nevada Range and Cascade Range, and the semi-arid eastern parts of Washington, Oregon, and southern California. Geographic outliers for counties classified as having *I. pacificus* established included four counties in western and central Utah, and a single county in northwestern Arizona. Tick collection locations in these two states, where most areas are too arid to support *I. pacificus*, typically were at high elevation (2000–2200 m) in scrub oak habitat (see Sections 3.5-3.6). Dennis et al. (1998) described the Arizona location as an isolated “sky island” where snow melt provided sufficient moisture and vegetation to support a tick population, and noted that narrow bands of riverine habitat in southern Utah may support *I. pacificus* in otherwise too arid environments.

Two decades later, Eisen et al. (2016a) updated the county-level distribution map making a minor modification to the “established” definition; established counties were defined by at least six individuals of a single life stage or at least two life stages detected in the county *within a single year*. County records included those from Dennis et al. (1998), with county status updated based on publications from 1996 through 2015, records from state health department websites, and reports from public health officials, acarologists, and Lyme disease investigators. There were very few (n=5) additional counties classified as having *I. pacificus* established from 1996 to 2015 (Table 1); these included four counties in western Washington and a single county in western Utah (Fig. 2).

The CDC then initiated a national program for surveillance of ticks and their associated pathogens in 2018 (Eisen and Paddock, 2021; CDC, 2023b). Data on *I. pacificus* are collated from state health departments and other partners, including the National Ecological Observatory Network (NEON, 2023), in ArboNET. There were no changes from 2016 to 2022 for counties classified as having *I. pacificus* established (CDC, 2023c; Table 1), though surveillance efforts were limited in the western US and counties are very large, potentially masking geographic changes at sub-county spatial scales. For those interested in records of *I. pacificus* from specific counties, Table 2 provides a breakdown of references for this tick species by state and county in the western United States. It also should be noted that Fleshman et al. (2021) presented a county level map for detection of *B. burgdorferi* s.s. in *I. pacificus* across the tick’s range, with similar information now provided by CDC in a regularly updated online map (CDC, 2023d).

With regards to presence of *I. pacificus*, the collection records from California, Oregon, and Washington presented in Sections 3.2 to 3.4 depict a wide distribution that has been largely stable since the 1930s. It seems likely the tick was then documented successively from local areas with suitable habitat as surveillance efforts were initiated over time. In contrast to *I. scapularis* in the eastern US (Eisen and Eisen, 2023), there is no clear evidence of range expansion for *I. pacificus* based on surveillance targeting questing ticks or from programs where ticks were recovered from wildlife, domestic animals, or humans. The density of nymphal or adult *I. pacificus* questing in a manner allowing for collection by drag/flag sampling (hereafter referred to simply as drag sampling) has been shown to vary between geographical areas as well as between habitat types (e.g., Eisen et al., 2006a, b; Swei et al., 2011a; Lane et al., 2013; MacDonald and Briggs, 2016). However, there is no evidence from long-term studies that the density of questing ticks has increased over time in specific areas. Based on the lack of clear evidence for either range expansion or local population increase of *I. pacificus* over the last half-century, it is not surprising that the incidence of reported Lyme disease cases has remained stable in the Pacific Coast states from the early 1990s, when Lyme disease became a notifiable condition, to present (<1 annual case per 100,000 population in California and Washington, and <2 annual cases per 100,000 population in Oregon; Bacon et al., 2008; Schwartz et al., 2017; CDC, 2023e). Other factors that could have influenced Lyme disease incidence over time include increased encroachment of human habitation on tick habitat and changes in the levels of human recreational activities in tick habitat or use of personal protection measures such as repellents.

### 3.2. Records from California

The California collection records for *I. pacificus* presented below are broken down into multiple time periods: 1893 to 1920 (Section 3.2.1.1), 1921 to 1950 (Section 3.2.1.2), 1951 to 1970 (Section 3.2.2.1), 1971 to 1984 (Section 3.2.2.2), 1985 to 1995 (Section 3.2.2.3), and 1996 to present (Section 3.2.3). Hui et al. (2004) noted there was no systematic active surveillance for *I. pacificus* in California by drag sampling until the mid-1980s, and that previous records in the CDPH database came from published literature (including records from Furman and Loomis, 1984), special research projects associated with surveillance for Q fever, Colorado tick fever, or plague, and ticks removed opportunistically from humans, domestic animals, and wildlife.

**Fig. 3.**
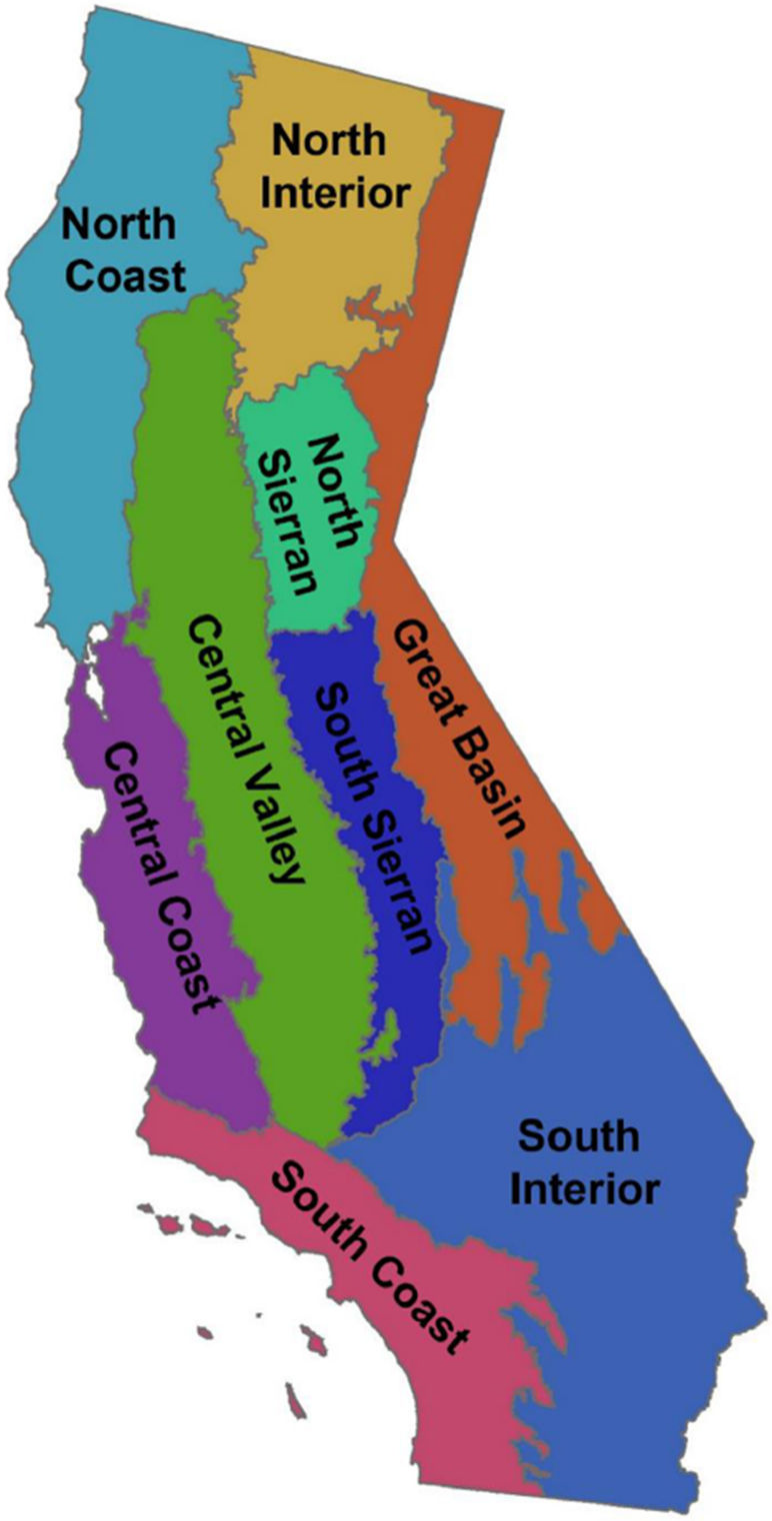
California vegetation mapping zones (adapted from USDA, 2023).

As California is climatically and ecologically diverse, with environments ranging from highly suitable to unsuitable for *I. pacificus*, we use broad geographic areas with more uniform environments (following vegetation mapping zones presented by the United States Department of Agriculture; USDA, 2023; see also Fig. 3) to guide the presentation of county level collection records within the state. Six mapping zones include substantial areas with habitat suitable for *I. pacificus*: 1) South Coast (from San Diego to Santa Barbara counties along the coast, including the Peninsular Range, Transverse Range, and the southern tip of the Southern Coast Range; and also containing the far western parts of inland Riverside and San Bernardino counties); 2) Central Coast (from San Luis Obispo to San Francisco counties along the coast, including the Southern Coast Range; and also containing all or portions of neighboring counties to the east up to the edge of the Central Valley); 3) North Coast (from Marin to Del Norte counties along the coast, including the Northern Coast Range; and also containing all or portions of neighboring counties to the east up to the edge of the Central Valley as well as counties or parts of counties located in the Klamath Mountains in the far north); 4) South Sierran (including the eastern parts of counties that extend from the Central Valley into the Sierra Nevada Range, from Kern County to the south to Calaveras County to the north); 5) North Sierran (including the eastern parts of counties that extend from the Central Valley into the Sierra Nevada Range, from Amador County to the south to Butte and Plumas counties to the north); and 6) North Interior (including parts of Tehama, Plumas, Shasta, Lassen, Siskiyou, and Modoc counties located in the Cascade Range). Three additional zones contain only sporadic areas with habitat suitable for *I. pacificus:* 7) Central Valley (including flat and predominantly agricultural parts of counties that also extend into the Coast Ranges to the west or Sierra Nevada Range to the east, from Kern County to the south to Shasta County to the north); 8) South Interior (primarily hot desert environments, including Imperial County and parts of San Diego, Los Angeles, Riverside, San Bernardino, Kern, and Inyo counties); and 9) Great Basin (primarily cold desert environments, including parts of Inyo, Mono, Lassen and Modoc counties in far eastern California). Suitable habitats for *I. pacificus* in these three zones may include riparian areas or, in the specific case of the Central Valley, isolated higher elevation areas such as the Sutter Buttes (Wright et al., 2003a, 2006).

For county records that do not include specific collection locations within the county, and where the county encompasses multiple vegetation mapping zones, we make the assumption that the ticks were recovered in the zone with more suitable habitat for *I. pacificus* for counties located partly in the South Interior, Central Valley, or Great Basin zones (low suitability for *I. pacificus*) and partly in the South Coast, Central Coast, North Coast, South Sierran or North Sierran zones (high suitability). Two counties merit special mention in this respect: Inyo County contains large portions characterized as South Interior and Great Basin vegetation zones but also a small portion of the more suitable South Sierran vegetation zone in the far west of the county; and Fresno County extends from the Central Coast through the Central Valley into the South Sierran vegetation zone. It also should be noted that records from California, as well as the other states presented in Sections 3.3 to 3.9, include collections with variable certainty for the source location being within the county from which *I. pacificus* ticks were recorded: high certainty for specimens collected by drag sampling or carbon dioxide traps, or recovered from wildlife with limited home ranges (such as rodents, insectivores, lagomorphs, and lizards); moderate certainty for specimens recovered from wildlife with more extensive home ranges (such as deer, carnivores, and birds); and lower certainty for specimens recovered from humans or domestic animals, in the absence of travel histories.

#### 3.2.1. California records from 1893 to 1950

Collection records for *I. pacificus* were plentiful from California during this early time period. Records from 1893 to 1920 almost exclusively involved adults, whereas records from the late 1920s onward included both adults and immatures.

##### 3.2.1.1. Records from 1893 to 1920.

Neumann (1896) included *I. pacificus* (=*I. ricinus*) as occurring in California, but without mentioning hosts, specific locations within the state, or collection years. The earliest detailed records representing *I. pacificus* were presented by Nuttall et al. (1911), who included collections from Santa Clara County, on the Central Coast, of *I. ricinus* from mouse in 1893 and mountain lion in 1894. Additional sources (Hui et al., 2004; CDPH, unpublished data) provide further details for the 1894 *I. pacificus* collections on the Central Coast: six males and seven females were recovered from a mountain lion in Santa Clara County, and two males from a mountain lion in San Benito County. There also are additional very early unpublished records for *I. pacificus* on the Central Coast, including in 1896 for a nymph collected from a bird in Santa Clara County and in 1900 for 12 adults from unknown hosts in Monterey County (CDPH, unpublished data).

Banks (1904) presented information for *I. pacificus* (as a new species named *I. californicus*) collected from a bird (*Toxostoma crissale;* Crissal thrasher) in Los Angeles County on the South Coast, but this particular record is considered to rather have been specimens of *Ixodes brunneus* (see Cooley and Kohls, 1943). Banks (1908) then reported collection of *I. pacificus* (= *I. californicus*) adults from fox and deer on the Central Coast (Santa Clara County and the “Santa Cruz Mountains”) and the North Coast (Humboldt County), but without specifying years of collection. However, both the CDPH database and the USNTC appears to include these records, with collection years of 1903 for a Humboldt County record of two females from deer, and 1907 for a Santa Cruz County record of one female and one male from an unknown host. These and later records of *I. californicus* are considered to represent *I. pacificus* (Cooley and Kohls, 1943; Guglielmone and Nava, 2014). There also is another early record of *I. pacificus* from the North Coast in 1906, when a nymph was recovered from a lizard in Mendocino County (CDPH, unpublished data).

Based on unpublished data from CDPH and USNTC, there was a suite of California collection records for *I. pacificus* adults from 1910 to 1915. Most of these records involved small numbers (up to five) of adults recovered from humans or domestic animals, but a few collections included more than 10 adults from domestic animals or deer. Collection locations were widespread, including counties on the South Coast (Los Angeles, Orange, San Bernardino, and San Diego), Central Coast (Alameda and Santa Clara), and North Coast (Humboldt, Lake, Mendocino, Siskiyou, Sonoma, and Trinity), as well as in the South Sierran zone (Madera) and North Sierran zone (El Dorado and Placer). Additionally, Clarke (1912, 1913) reported infestation by adult *I. pacificus* (=*I. ricinus* var. *californicus*) for nine deer (*O. columbianus columbianus*) in California from 1911 to 1912, but without specific information for collection locations. It was noted that some deer only carried a few ticks while others were infested by two to three dozen ticks.

##### 3.2.1.2. Records from 1921 to 1950.

Numerous additional California records for *I. pacificus* (including *I. ricinus californicus*) were presented in publications or included in the CDPH database or the USNTC for the time period from 1927 to 1950. Additionally, Boynton and Woods (1933) reported collection of *I. pacificus* (=*I. ricinus californicus*) from deer (*O. columbianus scaphiotus*) but without specifying the year and collection location. Collections from 1927 to 1950 included ticks from a wide range of sources, including adults collected by drag sampling or recovered from humans, domestic animals, or wildlife (primarily deer and carnivores), as well as immatures collected from lizards, rodents, and birds. Drag sampling often yielded large numbers of adults; for example, more than 250 adults were collected in a few hours from a single site in Monterey County in 1932 (Jellison, 1934).

South Coast records included collections of *I. pacificus* in western Riverside County from 1932 to 1935 (adults from drag sampling, dog, and lagomorph) and in 1949 (adults from deer); Santa Barbara County from 1932 to 1933 (adults from drag sampling and dog); Orange County from 1933 to 1937 and from 1947 to 1948 (adults from drag sampling and carnivore, and one nymph from drag sampling); western San Bernardino County in 1933 and 1942 (adults from domestic animals); Los Angeles County from 1935 to 1950 (adults from drag sampling, human, dog, and deer, and immatures from lizard, bird, rodent, and lagomorph); Ventura County in 1937 (adults from drag sampling and horse); and San Diego County in 1948 (one adult from drag sampling) (Jellison, 1934; Cooley and Kohls, 1945; Webb et al., 1990a; CDPH and USNTC, unpublished data).

Central Coast records of *I. pacificus* included collections in Alameda County from 1929 to 1933 (adults from drag sampling and dog, and immatures from birds) and 1936 to 1945 (adults from drag sampling, human, domestic animals, and rodent, and immatures from lizard, rodent, insectivore, carnivore, and bird); Monterey County from 1929 to 1932 (adults from drag sampling, and immatures from lizard) and 1938 to 1949 (adults from domestic animals, rodent, carnivore, and deer, and immatures from lizard and rodent); San Benito County in 1932 and 1937 (adults from drag sampling and cattle, and immatures from lizard and rodent); San Luis Obispo County from 1932 to 1943 (adults from drag sampling and cattle, and immatures from lizard); Santa Clara County in 1932 and 1948 (adults from drag sampling and domestic animals); Contra Costa County from 1937 to 1949 (adults from drag sampling and domestic animals, and immatures from lizard); San Francisco County from 1938 to 1946 (adults from human); San Mateo County in 1947 (adults from unknown source); and Santa Cruz County in 1949 (adults from dog) (Jellison, 1934; Cooley and Kohls, 1945; Holdenried et al., 1951, Linsdale and Tevis, 1951; Linsdale and Tomich, 1953; Webb et al., 1990a; CDPH and USNTC, unpublished data).

North Coast records included collections of *I. pacificus* in Sonoma County in 1932 (one adult from dog) and from 1949 to 1950 (immatures from lagomorph); Humboldt County from 1933 to 1948 (adults from drag sampling and domestic animals, and immatures from lizard); Lake County from 1933 to 1941 (adults from domestic animals and carnivore); Mendocino County from 1933 to 1937 (one adult from dog, and immatures from lizard); Napa County in 1933 (adults from human) and 1949 (immatures from lagomorph); Siskiyou County in 1933 (one adult from unknown source); Del Norte County in 1935 (adults from drag sampling and domestic animals); Marin County from 1938 to 1949 (adults from drag sampling, human, dog, deer, and carnivore, and immatures from lizard and rodent); Solano County in 1941 (immatures from lizard); and Trinity County in 1950 (adults from drag sampling) (Jellison, 1934; Kohls and Cooley, 1937; Cooley and Kohls, 1945; Webb et al., 1990a; CDPH and USNTC, unpublished data).

There also were records of *I. pacificus* from inland locations in California. In the South Interior zone, immatures were recovered from lizards in 1927 in the Imperial Valley, Imperial County (Webb et al., 1990a; CDPH, unpublished data). Records in the South Sierran zone included Madera and Tulare counties from 1932 to 1941 (adults from drag sampling, human, and domestic animals) (Jellison, 1934; Cooley and Kohls, 1945; CDPH and USNTC, unpublished data). Records in the North Sierran zone came from El Dorado and Plumas counties from 1949 to 1950 (adults from drag sampling, human, dog, and deer, and immatures from lizard and rodent) (Webb et al., 1990a; CDPH and USNTC, unpublished data). In the extreme northern end of the Central Valley, a nymph from rodent and two ticks of unknown life stage were recorded from Shasta County (including in Redding) from 1933 to 1934 (Kohls and Cooley, 1937; CDPH, unpublished data). Finally, there were records of adults from dog in Fresno County in 1947 (USNTC, unpublished data; not clear if this was in the Central Coast or South Sierran portion of Fresno County); and adults from bear in Kern County in 1947 (CDPH and USNTC, unpublished data; most likely in the South Sierran portion of Kern County).

#### 3.2.2. California records from 1951 to 1995

Collection records for *I. pacificus* from California continued to accumulate from 1951 to the early 1980s, and then increased dramatically in volume from the mid-1980s to mid-1990s. The overall geographic range remained largely unchanged over this time period but surveys in new areas within the range provided a more detailed picture of where the tick was present.

##### 3.2.2.1. Records from 1951 to 1970.

The majority of *I. pacificus* records from this time period are from the CDPH database (unpublished data), with additional records from the USNTC (unpublished data) and published literature. South Coast records came from Los Angeles County from 1951 to 1960 (adults from drag sampling, human, and lagomorph); Orange County from 1952 to 1967 (adults from horse, rodent, and carnivore); Riverside County from 1952 to 1970 (adults from carnivore); San Bernardino County from 1952 to 1964 (adults from drag sampling and carnivore, and one nymph from lizard); San Diego County from 1952 to 1963 (adults from drag sampling, domestic animals, and carnivore); Santa Barbara County from 1955 to 1970 (adults from human and domestic animals, and immatures from lizard); and Ventura County from 1961 to 1966 (adults from drag sampling, human, cattle, and rodent, and immatures from lizard) (Ryckman et al., 1955; Webb et al., 1990a; CDPH, unpublished data).

Central Coast records for *I. pacificus* included Alameda County from 1951 to 1970 (adults from drag sampling, human, domestic animals, and rodent, and immatures from cat and lizard); Monterey County from 1951 to 1970 (adults from drag sampling, human, domestic animals, and carnivore, and immatures from lizard); San Benito County from 1951 to 1970 (adults from drag sampling, deer, and carnivore); Santa Clara County from 1951 to 1967 (adults from horse, and one nymph from deer); San Mateo County from 1954 to 1963 (adults from human and horse, and immatures from lizard, rodent, and insectivore); Santa Cruz County from 1955 to 1970 (adults from human); Contra Costa County from 1956 to 1970 (adults from drag sampling, human, and domestic animals, and immatures from human and lizard); and San Luis Obispo County from 1963 to 1968 (adults from drag sampling and horse) (Osebold et al., 1962; Mohr et al., 1964; Webb et al., 1990a; CDPH, unpublished data). There also were *I. pacificus* adults taken from dog in Stanislaus County, most likely in the Central Coast portion of the county (CDPH, unpublished data), and *I. pacificus* ticks were collected with carbon dioxide traps in the San Francisco Bay area (Garcia, 1962).

North Coast records for *I. pacificus* included Mendocino County from 1951 to 1968 (adults from drag sampling and deer); Sonoma County from 1951 to 1970 (adults from drag sampling, human, and dog, and immatures from human and bird); Marin County from 1952 to 1970 (adults from drag sampling, human, and rodent, and immatures from human and rodent); Napa County from 1955 to 1958 (adults from human und unknown source); Humboldt County from 1959 to 1969 (adults from drag sampling, human, lagomorph, and carnivore, and immatures from human and rodent); Shasta County from 1959 to 1964 (adults from drag sampling); Siskiyou County from 1960 to 1970 (adults and one nymph from drag sampling and carbon dioxide traps); Solano County from 1962 to 1963 (adults from human and cattle); and Del Norte and Lake counties from 1968 to 1970 (adults from drag sampling) (Longhurst and Douglas, 1953; Osebold et al., 1962; Browning and Lauppe, 1964; CDPH and USNTC, unpublished data). There also were records from Colusa and Yolo counties, most likely in the North Coast portion of the counties, from 1959 to 1966 (adults from coyote, and immatures from lizard) (CDPH, unpublished data).

Additional records of *I. pacificus* came from the South Sierran and North Sierran zones, as well as the Central Valley and the North Interior zone. South Sierran records for *I. pacificus* included Madera County from 1953 to 1962 (adults from drag sampling and horse); Tulare County from 1959 to 1970 (adults from drag sampling, and one larva from lizard); Tuolumne County from 1961 to 1965 (adults from drag sampling); Kern County from 1962 to 1968 (adults from deer and unknown life stage from drag sampling); Calaveras County from 1963 to 1968 (adults from horse and deer); and the neighboring Mariposa and Merced counties from 1963 to 1965 (adults from dog) (Brunetti, 1965; CDPH, unpublished data). North Sierran records for *I. pacificus* included El Dorado County from 1951 to 1970 (adults from drag sampling, human, dog, deer, and carnivore, and immatures from rodent); Nevada County from 1951 to 1970 (adults from drag sampling, human, and dog); Plumas County from 1951 to 1968 (adults from drag sampling, and immatures from rodent); Butte County in 1960 (adults from human and carnivore); Placer County from 1960 to 1965 (adults from drag sampling); Amador County from 1964 to 1967 (adults from drag sampling); and Sierra County in 1964 (one adult from drag sampling) (Brunetti, 1965; CDPH, unpublished data).

There also were a few records of *I. pacificus* from Sacramento County in the Central Valley from 1963 (adults from dog), and one record from Lassen County in the North Interior zone in 1959 (one adult from drag sampling) (CDPH, unpublished data). Finally, there were records of adults from drag sampling and deer in Fresno County from 1961 to 1964 (CDPH, unpublished data; not clear if this was in the Central Coast or South Sierran portion of Fresno County).

Arthur and Snow (1968) summarized records for *I. pacificus* in California without specifying collection years or life stages and numbers of ticks recovered. This likely included both previously published and new records. Collection records for questing ticks included Los Angeles, Orange, and Riverside counties on the South Coast; Alameda, Monterey, and Santa Clara counties on the Central Coast; Humboldt and Del Norte counties on the North Coast; Madera County in the South Sierran zone; and Plumas County in the North Sierran zone. Additional records were included for ticks taken from various hosts in locations (indicated on a state map without county borders) representing multiple other counties along the Coast Ranges and the Sierra Nevada Range.

##### 3.2.2.2. Records from 1971 to 1984.

*Ixodes pacificus* records from this time period came from CDPH (unpublished data) together with published literature. South Coast records came from San Diego County from 1971 to 1972 (adults from unknown source, and immatures from rodent); Santa Barbara County from 1971 to 1981 (adults from drag sampling and carnivore); Ventura County in 1972 and 1980 (adults from dog); Los Angeles County in 1974 (adults from carnivore); and Orange County in 1984 (immatures from lizard) (Nelson, 1973; Webb et al., 1990a; CDPH, unpublished data). There also were records of *I. pacificus* from 1975 to the early 1980s on California Channel Islands assigned to Santa Barbara and Los Angeles counties (adults from human, dog, carnivore, and wild pig, and immatures from lizard and rodent) (Lane et al., 1982; Bennett, 1987; Webb et al., 1990a). Central Coast records for *I. pacificus* included Contra Costa County from 1971 to 1979 (adults from drag sampling, human, and domestic animals, and one nymph from human); Monterey County from 1971 to 1979 (adults from drag sampling and human, and immatures from lizard); Santa Cruz County from 1971 to 1978 (adults from dog and unknown source); San Mateo County from 1972 to 1976 (one adult from human, and immatures from rodent); San Francisco County in 1975 (one adult collected inside a house); and San Luis Obispo County from 1978 to 1981 (adults from human and carnivore) (Philip et al., 1981; CDPH, unpublished data).

North Coast records for *I. pacificus* notably included extensive collections from the Hopland Field Station in Mendocino County from 1971 to 1979, including all life stages taken from deer (*O. hemionus columbianus*; totals of 46 larvae, 26 nymphs, and more than 1300 adults), more than 200 immatures taken primarily from rodents and lagomorphs, and more than 150 adults collected from vegetation by drag sampling (Westrom, 1975; Lane et al., 1981; Philip et al., 1981; Westrom et al., 1985). Additional records for *I. pacificus* from the North Coast for the period from 1971 to 1981 included Lake County in 1971 (immatures from lizard); Marin County from 1971 to 1981 (adults from human and domestic animals, and immatures from human and bird); Siskiyou County from 1971 to 1974 (adults from drag sampling and dog); Sonoma County from 1971 to 1981 (adults from drag sampling, human, and dog, and immatures from drag sampling, rodent, and lizard); Napa County from 1972 to 1977 (adults from human, domestic animals, and dog); Mendocino County from 1972 to 1980 (adults from human, dog, and carnivore); Trinity County from 1973 to 1976 (adults from human and unknown source); Humboldt County from 1979 to 1981 (adults from drag sampling and rodent, and one nymph from human); and Yolo County, most likely in the North Coast portion of the county, in 1981 (one adult from cattle) (Botzler et al., 1980; CDPH, unpublished data).

Following the discovery in 1981 that *I. scapularis* in the eastern US were infected with the causative agent of Lyme disease (Burgdorfer et al., 1982), hundreds of adult *I. pacificus* were collected by drag sampling from 1982 to 1984 in Marin, Mendocino, and Sonoma counties on the North Coast to be examined for presence of this disease agent (Burgdorfer et al., 1985). This study provided the first documentation of Lyme disease spirochetes from *I. pacificus* in California, with infected ticks collected from all three counties albeit at low prevalence of infection (0.9–1.4%). It also should be noted that Naversen and Gardner (1978) reported a case of erythema chronicum migrans from Sonoma County on the North Coast in the late 1970s (the year was not specified) associated with the bite of a tick determined to belong to the genus *Ixodes*, most likely *I. pacificus*.

There also were records of *I. pacificus* from the South Sierran and North Sierran zones, as well as the Central Valley and the North Interior zone. South Sierran records for *I. pacificus* included Kern County in 1971 (one adult and one nymph from rodent); Tulare County from 1971 to 1980 (adults from drag sampling, human, dog, and bear, and immatures from lizard); Tuolumne County in 1973 (one adult from drag sampling); and Mariposa County from 1975 to 1978 (adults from bear) (Furman and Loomis, 1984; Webb et al., 1990a; CDPH, unpublished data). North Sierran records for *I. pacificus* included Placer County from 1974 to 1978 (adults from drag sampling); and Amador and Sierra counties in 1973 (adults from human) (Webb et al., 1990a; CDPH, unpublished data). Additional records came from Sacramento County in the Central Valley in 1974 (immatures from lizard); Lassen County in the North Interior zone in 1977 (one adult from human); and Inyo County in 1977 (one adult from elk) (Webb et al., 1990a; CDPH, unpublished data).

Furman and Loomis (1984) then provided a summary of collection locations for *I. pacificus* in California. The geographic distribution of collection locations was visualized as points on a state map. The specific counties and collection years these point locations represented were unfortunately not specified in the publication, although records specifically for the larval stage later were included in the publication by Webb et al. (1990a) on the larval ticks of the genus *Ixodes* in California. Furman and Loomis (1984) noted that *I. pacificus* had been recorded from 50 of the 58 counties in California, in locations ranging in elevation from sea level to over 2150 m. The distribution map for *I. pacificus* included numerous point locations in the South Coast zone (>30 locations), Central Coast zone (>30 locations), North Coast zone (>50 locations), South Sierran zone (approximately 15 locations), and North Sierran zone (approximately 10 locations). Lower numbers of collection point locations were indicated as falling within the Central Valley zone (approximately five locations in the central and northern parts of the Central Valley), North Interior zone (three locations in the southern part of the zone), Great Basin zone (one location situated in extreme eastern El Dorado County or Placer County on the Nevada border and another location in the northeastern part of Inyo County), and South Interior zone (one location in Imperial County).

##### 3.2.2.3. Records from 1985 to 1995.

Collection records from field studies specifically targeting *I. pacificus* accumulated rapidly from the mid-1980s onward. From the mid-1980s to the mid-1990s, large numbers of *I. pacificus* ticks were collected by drag sampling or from wildlife (lizards, rodents, birds, lagomorphs, and deer) across California (Lane and Burgdorfer, 1986,^1987^,^1988^; Bissett and Hill, 1987; Lane and Lavoie, 1988; Loye and Lane, 1988; Lane and Loye, 1989, 1991; Lane and Pascocello, 1989; Lane, 1990a, 1990b, 1990c, 1992, 1996; Lane and Stubbs, 1990; Manweiler et al., 1990, 1992; Webb et al., 1990a, 1990b, 1992; Goldberg and Bursey, 1991; Boyce et al., 1992; Brown and Lane, 1992; Lane et al., 1992; Meyers et al., 1992; Monsen et al., 1990, 1992; Wright, 1992; Kramer and Beesley, 1993; Olson et al., 1993; Schwan et al., 1993; Clover and Lane, 1995; Barlough et al., 1997; Castro et al., 1997; Peavey et al., 1997; Hui et al., 1998, 2004; Lang, 1999; Casher et al., 2002; CDPH, unpublished data). During this time period, *I. pacificus* was collected from all counties falling entirely or partly within the South Coast zone (Los Angeles, Orange, Riverside, San Bernardino, San Diego, Santa Barbara, and Ventura), all counties falling entirely or partly within the Central Coast zone (Alameda, Contra Costa, Fresno, Kings, Merced, Monterey, San Benito, San Luis Obispo, San Mateo, Santa Clara, Santa Cruz, and Stanislaus), and all counties falling entirely or partly within the North Coast zone (Colusa, Del Norte, Glenn, Humboldt, Lake, Marin, Mendocino, Napa, Shasta, Siskiyou, Solano, Sonoma, Tehama, Trinity, and Yolo). Collections of *I. pacificus* also were made from all counties located partly in the South Sierran zone (Calaveras, Fresno, Kern, Madera, Mariposa, Tulare, and Tuolumne) and the North Sierran zone (Amador, Butte, El Dorado, Nevada, Placer, Plumas, Sierra, and Yuba). There also were notable records of *I. pacificus* from San Joaquin, Sacramento, and Sutter counties in the Central Valley (Wright, 1992; Wright et al., 1996; CDPH, unpublished data), the South Interior portion of San Bernardino County (Schwan et al., 1993), and Lassen County in the North Interior zone (CDPH, unpublished data). Finally, there was a record of adults collected by drag sampling from Inyo County in 1989 (CDPH, unpublished data). Negative data included drag sampling efforts failing to produce *I. pacificus* in Alpine County in the South Sierran zone, Mono County in the Great Basin zone, and Modoc County in the North Interior zone (Schwan et al., 1993).

#### 3.2.3. California records from 1996 to present

Dennis et al. (1998) listed 55 of 58 counties in California as having established populations of *I. pacificus* by 1996. One county was classified as *I. pacificus* being reported (Mono County, in the Great Basin zone with the westernmost portion of the county extending down into the South Sierran zone) and two counties had no reports of this tick (Modoc County in the North Interior zone and Alpine County in the South Sierran zone) (Fig. 2). No change to county classifications for *I. pacificus* in California has occurred from 1996 to 2022 (Eisen et al., 2016a; CDC, 2023c; Fig. 2).

Numerous field studies resulting in collection of *I. pacificus* have been conducted from the late 1990s to present. Focal areas for these studies have included: (i) the Marin-Napa-Yolo-Sonoma-Mendocino-Humboldt section of the North Coast (Reubel et al., 1998; Foley et al., 1999, 2007, 2011; Kramer et al., 1999; Nicholson et al., 1999; Vredevoe et al., 1999; Li et al., 2000; Tälleklint-Eisen and Eisen, 1999; Tälleklint-Eisen and Lane, 1999, L. 2000; Eisen et al., 2001, 2002, 2003, 2004a, 2004b, 2004c, 2004d, 2006a, 2006b; Lane et al., 2001, 2005a, 2005b, R.S. 2006, 2007, 2010; Slowik and Lane, 2001; Wright et al., 2003b, 2010, 2011, S.A. 2012; Fritz et al., 2005; Nieto and Foley, 2008, 2009; Salkeld et al., 2008, 2014a, 2014b, 2015, 2021; Gabriel et al., 2009; Nieto et al., 2009, 2010; Castro and Clover, 2010; Foley and Nieto, 2011; Phan et al., 2011; Rejmanek et al., 2011; Swei et al., 2011a, 2011b, 2011c, A. 2012, 2015; Dingler et al., 2014; Foley and Piovia-Scott, 2014; Eshoo et al., 2015; Bonkrude, 2016; Nieto and Salkeld, 2016; Ryan et al., 2016; Lilly et al., 2022; Paddock et al., 2022); (ii) the Alameda-San Mateo-Santa Clara-Santa Cruz-Monterey-San Luis Obispo section of the Central Coast (Foley et al., 1999, 2011; Kramer et al., 1999; Chang et al., 2001; Holden et al., 2003, K. 2006; Nieto et al., 2007; Lumbad et al., 2011; Padgett and Bonilla, 2011; Rejmanek et al., 2011; Fedorova et al., 2014; Foley and Piovia-Scott, 2014; Salkeld et al., 2014a, 2014b, 2015, 2021; Eshoo et al., 2015; Bonilla, 2016; MacDonald et al., 2016; Nieto and Salkeld, 2016; Kirkpatrick et al., 2017; Kleinjan et al., 2019; Crews and Roth, 2020; López-Pérez et al., 2021; Nakano et al., 2021; James et al., 2022; Paddock et al., 2022); (iii) Los Angeles, Orange, Riverside, and Santa Barbara counties on the South Coast (Crooks et al., 2001, 2004; Hu et al., 2004a, b; Lane et al., 2013, Eshoo et al., 2015; MacDonald and Briggs, 2016; Billeter et al., 2017; MacDonald, 2018; MacDonald et al., 2017, 2020a); (iv) El Dorado, Fresno, Madera, Mariposa, Nevada, Placer, Tehama and Yuba counties in the Sierran zones (Reubel et al., 1998; Wright et al., 1998, S.A. 2000, 2003b; Foley et al., 1999, 2011, 2016; Kramer et al., 1999; Vredevoe et al., 1999; Fleer et al., 2011; Osborne et al., 2020; Hacker et al., 2021; Paddock et al., 2022); and (v) Sacramento and Sutter counties in the Central Valley (Wright et al., 2003a, 2003b, S.A. 2006; Foley et al., 2011). Hui et al. (2004) also noted collection of two *I. pacificus* nymphs in 2003 during a relapsing fever case investigation in Mono County, which is located in the Great Basin zone with the western edge of the county extending into the South Sierran zone.

A few studies also have presented statewide county level data for collection of *I. pacificus* based on drag sampling (Padgett et al., 2014; Rose et al., 2019) or submission of ticks from community members (Salkeld et al., 2019; Porter et al., 2021). The counties with records of *I. pacificus* in these recent statewide studies are detailed in Table 2. However, it should be noted that drag sampling clearly demonstrates local presence of the tick whereas collection of ticks from community members or their pets may represent exposures occurring outside the county of residence unless there is a documented travel history. Padgett et al. (2014) and Rose et al. (2019) also presented data for infection with *B. burgdorferi* s.l. in *I. pacificus* nymphs and adults collected across California. Infected ticks were recorded across California, although it was noted that the prevalence of infection was much lower in southernmost California than in the central and northern parts of the state. There also was a notable collection of four *I. pacificus* ticks from gray foxes in Ensenada, Baja California, Mexico (near the US border) from 2017 to 2018 (López-Pérez et al., 2020).

#### 3.2.4. California record summary

The collection records for *I. pacificus* in California outlined above give a clear impression of the tick being widely distributed across the state in the early 1900s. The tick had already been recorded from 18 (31%) of California’s 58 counties by 1920, and from 34 (59%) counties by 1950, with a distribution spanning the entire coastline from San Diego County in the south to Del Norte County in the north and with sporadic collections from inland counties, ranging from Imperial County in the south to Plumas and Shasta counties in the north. County records often included both immatures and adults of *I. pacificus*, recovered from a wide range of sources including drag sampling or collection from humans, dogs, cats, horses, cattle, rodents, insectivores, lagomorphs, carnivores, deer, lizards, and birds. By 1984, *I. pacificus* had been documented from 50 (86%) counties in California, and this rose to 56 (97%) counties by 1996. There is no clear evidence of range expansion for *I. pacificus* in California based on surveillance targeting questing ticks or from programs where ticks were recovered from wildlife, domestic animals, or humans. Rather than reflecting true range expansion, changes in the documented geographic distribution of *I. pacificus* in California since the 1950s more likely in most cases resulted from intensified interest in this tick over time, leading to field studies in counties and local areas where no previous efforts had been made to collect *I. pacificus*. Table 2 provides a breakdown of references for *I. pacificus* records by county in California.

### 3.3. Records from Oregon

Oregon is ecologically diverse, with suitable environments for *I. pacificus* located primarily in the western, cooler and wetter part of the state (including the Coast Range, Willamette Valley, and Cascade Range) and along the Columbia River and its tributaries on the border with Washington in the north. The arid landscape in the southern and central parts of eastern Oregon is generally too dry for *I. pacificus* to persist. Collection records for *I. pacificus* in Oregon presented below are broken down into two different time periods: 1893 to 1950 (Section 3.3.1) and 1951 to present (Section 3.3.2).

#### 3.3.1. Oregon records from 1893 to 1950

The first records of *I. pacificus* in Oregon come from the US National Tick Collection (unpublished data), which includes collections in 1910 of more than 20 adults recovered from dogs or horses in Douglas and Josephine counties in the southwest and Lincoln County in the west-central part of the state. The next suite of records for *I. pacificus* (including *I. ricinus californicus*) came from 1932 to 1942, when the tick was recorded from 10 Oregon counties: Curry, Douglas, Jackson, and Josephine in the southwest; Benton and Lane in the west-central part of the state; Clackamas, Columbia, and Multnomah in the northwest; and Umatilla in the northeast (Kohls and Cooley, 1937; Cooley and Kohls, 1945; USNTC, unpublished data). This included adults collected by drag sampling or recovered, in most cases, from humans or domestic animals, as well as immatures taken from wild animals, including lizards. Most collection records included less than 10 immatures or adults but larger numbers of specimens (10 to 40 adults) were recovered by drag sampling in some cases. The outlier record in the northeast for Umatilla County, which borders on the Columbia River, was based on a single female collected from a human, with no travel history provided to rule out that the tick may have been acquired elsewhere. Additionally, Chamberlin (1937) presented maps for tick records in Oregon, without specific information for collection year, host, or life stages and numbers of ticks recovered, but indicating the counties where collections were made. Separate records were shown for *I. ricinus* and *I. californicus*. Collectively, this included records from nine counties in the western part of Oregon (Benton, Clackamas, Columbia, Curry, Douglas, Jackson, Josephine, Lincoln, and Multnomah) and one county in the northeast (Morrow, which is located along the Columbia River and borders on Umatilla County). The US National Tick Collection (unpublished data) also includes records from the late 1940s for more than 30 *I. pacificus* adults collected from vegetation, humans or domestic animals in Coos, Douglas, and Jackson counties in southwestern Oregon.

### 3.3.2. Oregon records from 1951 to present

Arthur and Snow (1968) presented records for *I. pacificus* in Oregon without specifying collection years or life stages and numbers of ticks recovered. The records included questing ticks collected in Curry, Douglas, and Josephine counties in the southwest and Hood River County in the northwest, together with ticks taken from various hosts in locations (indicated on a state map without county borders) representing multiple other counties along the Coast Range. The US National Tick Collection (unpublished data) does not include any records of *I. pacificus* from Oregon in the 1950s but there are a few records from 1965 for immatures taken from lizards in Benton and Douglas counties. This was followed by extensive *I. pacificus* records from Oregon based on surveys (primarily drag sampling for adults but in some cases also examination of rodents and lizards for immature ticks) conducted in recreational areas throughout the state from 1967 to 1979, together with previously unpublished records from the Rocky Mountain Laboratory (Easton, 1972; Easton and Goulding, 1974; Hughes et al., 1976; Easton et al., 1977; USNTC, unpublished data). These efforts yielded more than 4000 *I. pacificus* ticks from 20 counties, including 17 counties in western Oregon (Benton, Clackamas, Clatsop, Columbia, Coos, Curry, Douglas, Jackson, Josephine, Lane, Lincoln, Linn, Marion, Multnomah, Polk, Tillamook, and Washington) and three counties in northernmost central Oregon located along the Columbia River (Hood River, Sherman, and Wasco). Collection efforts in counties in eastern Oregon commonly yielded *Dermacentor andersoni* (the Rocky Mountain wood tick) but no *I. pacificus* (Easton et al., 1977). Following the detection in 1981 of Lyme disease spirochetes in *I. scapularis* in the eastern US (Burgdorfer et al., 1982), *I. pacificus* adults were collected from Douglas and Josephine counties in southwestern Oregon from 1982 to 1984 and similarly found to be infected (Burgdorfer et al., 1985). A follow-up study where *I. pacificus* adults were collected in Jackson and Josephine counties in southwestern Oregon in 1997 confirmed that spirochetes infecting these ticks were *B. burgdorferi* s.s., with an infection rate of 3% (Burkot et al., 1999). Moreover, it was shown that *I. pacificus* immatures, especially larvae, commonly fed on rodents (*Neotoma* woodrats and *Peromyscus* mice) found to be infected with *B. burgdorferi* s.s.

Dennis et al. (1998) listed 18 counties in western and north-central Oregon as having established populations of *I. pacificus* (Fig. 2) and four counties with the tick reported (Columbia and Polk in the west, Jefferson in the north-central part of the state, and Umatilla in the northeast). The only county mentioned above as having a record of *I. pacificus* but not included by Dennis et al. (1998) is Morrow County in northeastern Oregon. No change to county classifications for *I. pacificus* being established in Oregon occurred from 1996 to 2022 (Eisen et al., 2016a; CDC, 2023c). Other notable publications from Oregon include Doggett et al. (2008) and Porter et al. (2021). These publications provided records of *I. pacificus* adults, collected by drag sampling from 2003 to 2004 (Doggett et al., 2008) or recovered from humans or domestic animals from 2016 to 2017 (Porter et al., 2021), from areas where the tick was previously known to be established in western or north-central Oregon. Notably, this included reports of *I. pacificus* from Yamhill County in northwestern Oregon (Doggett et al., 2008; Porter et al., 2021), where the tick had been assumed to be present based on records from neighboring counties (Easton et al., 1977).

#### 3.3.3. Oregon record summary

The collection records for *I. pacificus* in Oregon outlined above indicate a wide distribution in the early 1900s across the western, environmentally favorable portion of the state (including the Coast Range, the Willamette Valley, and the foothills of the Cascade Range). *Ixodes pacificus* had already been recorded from 11 (58%) of the 19 counties located in this part of Oregon by 1950, with tick records spanning counties located on the California border in the south to the Washington border in the north. Following expanded tick surveillance efforts across Oregon from 1967 to 1979, *I. pacificus* had been recorded from 18 (95%) of the 19 western counties by 1980, as well as from counties further east along the Columbia River on the Washington border. Similar to California, there is no clear evidence of range expansion for *I. pacificus* in Oregon based on surveillance targeting questing ticks or from programs where ticks were recovered from wildlife, domestic animals, or humans. Table 2 provides a breakdown of references for *I. pacificus* records by county in Oregon.

### 3.4. Records from Washington (and British Columbia in Canada)

Washington is ecologically diverse, with suitable environments for *I. pacificus* located primarily in the western, cooler and wetter part of the state (including the Coast Range and Cascade Range) and along the Columbia River and its tributaries along the border with Oregon in the south. The arid landscape in the central and northern parts of eastern Washington is generally too dry for *I. pacificus* to persist. Collection records for *I. pacificus* in Washington presented below are broken down into two different time periods: 1893 to 1950 (Section 3.4) and 1951 to present (Section 3.4.2).

#### 3.4.1. Washington records from 1893 to 1950

*Ixodes pacificus* (=*I. ricinus californicus*) was first recorded from Washington in the early 1930s, when eight adults were recovered in 1930 from humans in Lewis County in the southwestern part of the state, and a single adult was taken in 1932 from a horse on Orcas Island in San Juan County in the northwest (Cooley and Kohls, 1945; USNTC, unpublished data). Additional records came in the late 1930s, when a female tick was recovered in 1936 from an unknown host in Pierce County in the west-central part of the state; six adults were recovered in 1939 from humans in Skagit County in the northwest; and four nymphs were taken in 1937 from lizards in Klickitat County in the south-central part of the state and in Pierce County (Cooley and Kohls, 1945; USNTC, unpublished data). The US National Tick Collection (unpublished data) also includes records from the early 1940s, from Clark County in the southwestern part of the state and Whatcom County in the northwest, most often including single females recovered from humans (USNTC, unpublished data). It also should be noted that *I. pacificus* was occasionally recorded from the southern coast of British Columbia in Canada (just north of Washington State) from 1907 to 1929 (Nuttall et al., 1911; Gregson, 1956), and that extensive collections of this tick (=*I. ricinus californicus* or *I. californicus*) were made during the 1930s and 1940s in southwestern British Columbia (Gregson, 1935, 1942, 1956; Hearle, 1938; Cowan, 1946).

#### 3.4.2. Washington records from 1951 to present

Records of *I. pacificus* from the 1950s and early 1960s in western Washington included adult ticks recovered from humans or dogs in Clark, Klickitat, and Mason counties, and immatures taken from lizards in Klickitat County (USNTC, unpublished data). There also is a record of an *I. pacificus* tick recovered at a quarantine station in Hawaii from a dog traveling from Seattle (King County) in 1961 (Garrett and Haramoto, 1967). Arthur and Snow (1968) then presented records for *I. pacificus* in Washington without specifying collection years or life stages and numbers of ticks recovered. The records included questing ticks collected in King and Whatcom counties in the northwest, together with ticks taken from various hosts in locations (indicated on a state map without county borders) representing multiple counties along the Columbia River in the southwest (Clark, Skamania, and Klickitat based on map-indicated locations) and in the Puget Sound area in the northwest (Kitsap, Mason, Pierce, and Skagit based on map-indicated locations). The US National Tick Collection (unpublished data) also includes records from 1972 of more than 100 adult *I. pacificus* recovered from various hosts, including dogs and cats, in San Juan County in the northwest.

We are not aware of any further records of *I. pacificus* from Washington until 1996 when Dennis et al. (1998) listed 12 counties in the western part of the state as having established populations of *I. pacificus* (Chelan, Clark, Jefferson, Klickitat, Lewis, Mason, Pierce, San Juan, Skagit, Skamania, Thurston, and Whatcom; see Fig. 2), and another five western counties with the tick reported (Cowlitz, Island, King, Kitsap, and Snohomish). Based on unpublished data generated by the Washington State Department of Health, Eisen et al. (2016a) reclassified Clallam, Cowlitz, King, and Kitsap as counties with *I. pacificus* established (Fig. 2) and added four western counties as having *I. Pacificus* reported (Kittitas, Okanogan, Pacific, and Yakima). Dykstra et al. (2020) then reported results specifically for drag sampling for *I. pacificus* in western Washington from 2011 to 2016, with ticks recovered from seven counties (Clallam, Jefferson, Klickitat, Mason, Pierce, Thurston, and Yakima) and *B. burgdorferi* s.s. detected from 4% of tested adult ticks. Porter et al. (2021) provided records of *I. pacificus* recovered infesting humans or domestic animals, without documented travel histories, from 2016 to 2017 in 11 counties in western Washington (Chelan, Clallam, Grays Harbor, King, Kitsap, Klickitat, Lewis, Mason, Skamania, Thurston, and Yakima) and Pend Oreille County in far northeastern Washington. As the tick has not been collected by drag sampling from Pend Oreille County in northeastern Washington, we caution that the sole record of *I. pacificus* from this county (which represents a geographical outlier from the known distribution of the tick in Washington; see Figs. 1-2) may have resulted from travel exposure. By 2022, there were no changes from Eisen et al. (2016a) for counties in Washington classified as having established populations of *I. pacificus* (CDC, 2023c). Finally, it also should be noted that *I. pacificus* was commonly recorded from British Columbia in Canada from 1993 to 2018: the vast majority of collections came from southwestern coastal areas but there were occasional records also inland to the southeast and further north along the coast (Mak et al., 2010; Morshed et al., 2021).

#### 3.4.3. Washington record summary

Surveillance for *I. pacificus* was more limited throughout the 1900s in Washington compared to California and Oregon. Nevertheless, the collection records for *I. pacificus* outlined above indicate a wide distribution in the early 1900s across the environmentally favorable western portion of the state (including the Coast Range, the Puget Sound area, and the foothills of the Cascade Range). *Ixodes pacificus* was recorded from 7 (35%) of the 20 counties located in western Washington by 1950, with tick records spanning counties located on the Oregon border in the south to the border with Canada in the north. The number of western counties with records of *I. pacificus* then rose to 10 (50%) by 1980 and 17 (85%) by 1996. More intensive active surveillance for *I. pacificus* was not initiated until after 2010 in Washington. Similar to California and Oregon, there is no clear evidence of range expansion for *I. pacificus* in Washington based on surveillance targeting questing ticks or from programs where ticks were recovered from wildlife, domestic animals, or humans. Table 2 provides a breakdown of references for *I. pacificus* records by county in Washington.

### 3.5. Records from Utah

Utah has a semi-arid to arid climate where *I. pacificus* can persist only locally in especially moist environments. The first records of *I. pacificus* (=*I. ricinus californicus*) from Utah came in 1938: a total of three female ticks were recovered from two deer hunters in Beaver and Piute counties in the southwestern part of the state (Bishopp and Trembley, 1945). This was followed in 1945 by an *I. pacificus* tick recovered from a human in Millard County in west-central Utah (Edmunds, 1951). Additional collections were made from the 1950s to the early 1960s in western and central Utah. *Ixodes pacificus* adults were recovered from deer, dog, and human in Juab and Utah counties; and immatures were taken from rodents and a bird nest in Juab, Tooele, Utah, and Washington counties (Beck, 1955; Allred et al., 1960; Marchette et al., 1962; Johnson, 1966; USNTC, unpublished data). Arthur and Snow (1968) then presented records for *I. pacificus* in Utah without specifying collection years or life stages and numbers of ticks recovered. This included records of ticks taken from various hosts in locations (indicated on a state map without county borders) representing multiple counties in the western and central parts of the state.

To the tally of seven counties with collection records of *I. pacificus* by 1955 outlined above, Dennis et al. (1998) added only a single county (Salt Lake County in central Utah) by 1996. Davis et al. (2015) then conducted drag sampling for *I. pacificus* adults from 2011 to 2013 in 157 sites spread across nine counties in western and central Utah. One site in Tooele County produced more than 100 adults, whereas six other sites in Millard, Tooele, or Washington counties each yielded one to two adults, and no *I. pacificus* were collected from the remaining 150 sites. None of 119 examined *I. pacificus* adults were found to be infected with *B. burgdorferi* s.s. Sites with *I. pacificus* adults collected were typically at high elevation (2000–2200 m), with habitats comprised of scrub oak, brush, juniper, and grasses. Eisen et al. (2016a) included the same eight counties as Dennis et al. (1998) with records for *I. pacificus*, with five of these counties listed as having established tick populations (Juab, Salt Lake, Tooele, Utah, and Washington; see Fig. 2). No change to county classifications for *I. pacificus* occurred from 2016 to 2022 (CDC, 2023c). Table 2 provides a breakdown of references for *I. pacificus* records by county in Utah.

### 3.6. Records from Arizona

Arizona has a semi-arid to arid climate where *I. pacificus* can persist only locally in especially moist environments. The only county in Arizona with records of *I. pacificus* is Mohave in the northwestern part of the state (Olson et al., 1992; Table 2). Mohave County borders on Washington County in southwestern Utah where *I. pacificus* has been collected at high elevation in mountainous areas (Davis et al., 2015). In October 1991, two *I. pacificus* adults were recovered from humans after they hiked at high elevation (≈2200 m) in Hualapai Mountain County Park in Mohave County (Olson et al., 1992). Follow-up drag sampling in April of 1992 along the trail they had hiked yielded 65 adult *I. pacificus* collected from grass on south-facing slopes in a scrub oak habitat. Additional efforts in May and June of 1992 produced both adults (n=19) from drag sampling and immatures (n=17) from examination of lizards. Consequently, Mohave County has been listed as having an established population of *I. pacificus* (Dennis et al., 1998; Eisen et al., 2016a; CDC, 2023c; Fig. 2). However, to the best of our knowledge, this area has not been re-examined for presence of *I. pacificus* since 1992, and no efforts to collect *I. pacificus* have been made in other high elevation areas in northern Arizona. The adult ticks collected in 1992 also were examined for presence of spirochetes using a monoclonal antibody (H5332) reactive with *B. burgdorferi* s.s. (Olson et al., 1992). Two of the adults were found to be infected but based on the detection methodology it cannot be ruled out they were infected with another spirochete species within the *B. burgdorferi* s.l. complex or with *B. miyamotoi*.

### 3.7. Records from Nevada

Nevada has a semi-arid to arid climate where *I. pacificus* may persist only locally in especially moist environments. The only records of *I. pacificus* from Nevada came from 1959 to 1962 from the Nevada Test Site, which spans portions of Clark, Lincoln, and Nye counties in the far south of the state (Beck et al., 1963; Table 2). Clark County and Lincoln County borders on Mohave County in Arizona and Washington County in Utah, respectively, where *I. pacificus* is considered to be established (see Sections 3.5 and 3.6). Records of *I. pacificus* from the Nevada Test Site are limited to immatures (12 larvae and 1 nymph) taken from four pocket mice (Beck et al., 1963; see also Arthur and Snow, 1968). The specific collection locations for these pocket mice within the Nevada Test Site is not clear from the suite of publications on the overall survey of plants and animals for Nevada Test Site (Allred et al., 1963a, b; Beck et al., 1963). However, it was noted by Beck et al. (1963) that the larvae came from mice trapped in a brush habitat. We are not aware of any effort to collect *I. pacificus* from the Nevada Test Site after 1962, or to conduct a survey for this species in any other area in Nevada. To date, *I. pacificus* is classified as reported in two Nevada counties: Clark and Lincoln (Dennis et al., 1998; Eisen et al., 2016a; CDC, 2023c).

### 3.8. Records from Idaho

The only record of *I. pacificus* from Idaho is a female tick taken from a house cat in Pocatello, Bannock County, in the southeastern part of the state in 1970 (Easton et al., 1977; USNTC, unpublished data). There was no mention of whether the cat had a travel history. Bannock County is located close to the border with Utah, approximately 250 km north of Salt Lake County where *I. pacificus* is established. This county record was not included by Dennis et al. (1998) or Eisen et al. (2016a), but it does seem possible that local populations of *I. pacificus* could exist in southern Idaho.

### 3.9. Records from Alaska

*Ixodes pacificus* occurs commonly on the south coast of British Columbia in Canada, and there are a few records of this tick further north along the coast near the border with Alaska (Mak et al., 2010; Morshed et al., 2021). It therefore is not inconceivable that *I. pacificus* could be found in coastal areas in southernmost Alaska, such as in Ketchikan, Sitka or Juneau just to the north of British Columbia. Hahn et al. (2020) reported two female *I. pacificus* taken in 2017 and 2019 from dogs (with no recent travel history) in Anchorage, which is located in Anchorage Borough (County) on the coast in south-central Alaska. Surveillance for *I. pacificus* in southernmost coastal Alaska near the border with British Columbia is of interest.

## 4. Association of changing landscapes and host populations with the geographic distribution of *I. pacificus*

As noted by Eisen and Eisen (2023), dramatic increases over the last century in the geographic distribution of *I. scapularis* in the northern part of the eastern US likely resulted in large part from processes of reforestation (following deforestation in the 1800s) and, especially, population recovery of the white-tailed deer, *O. virginianus* (following decimation of deer leading to historically low populations around 1900, including virtual absence of deer from many areas in the Upper Midwest, Ohio Valley region, and the Northeast). The scenario that played out for *I. pacificus* in the far western US over the last century appears to be different in several respects.

Later colonization by European settlers of the Pacific Coast states, compared to states along the Eastern Seaboard, together with increasing awareness in the early 1900s of human impacts on the landscape resulted in more limited deforestation during the late 1800s and early 1900s within the geographic range of *I. pacificus* in the far west than in the *I. scapularis* range in the east (Li et al., 2023). Moreover, *I. pacificus* is present in a wide range of habitat types in the far western US. The tick occurs in densely or sparsely forested areas as well as in open settings dominated by brush or grass (Lane, 1990c; Lane and Stubbs, 1990; Kramer and Beesley, 1993; Hui et al., 1998; Tälleklint-Eisen and Eisen 1999; Eisen et al., 2006b; Swei et al., 2011; Lane et al., 2013; Salkeld et al., 2015; MacDonald et al. 2017). Due to its broad habitat associations, *I. pacificus* is not expected to be very sensitive to changes in the landscape over time, unless there also is a major impact on key host species.

The influx of settlers to the Pacific Coast states in the mid-1800s, motivated by the “gold rush”, resulted in strong, unregulated hunting pressure on deer that persisted until the turn of the century (Longhurst et al., 1952; Jensen et al., 2023). In response, hunting was increasingly regulated from the early 1900s onward in order to manage deer populations (Mackie, 1987; Jensen et al., 2023). Webb (2016) noted that the recovery of deer populations in the far western US was faster than for populations of white-tailed deer in the east because surviving deer populations in the early 1900s in the Pacific Coast states were generally higher in numbers (and presumably more geographically widespread within their range). Within the core geographic range of *I. pacificus*, Jensen et al. (2023) suggested that around 1920 there were more than 210,000 deer (*O. hemionus columbianus*) present in the Coast Ranges from northern California to southern British Columbia, together with an unspecified number of deer (*O. hemionus hemionus*) in the Coast Range of southern California and the Sierra Nevada foothills. Deer hunt data from California indicate annual harvests in the range of 25,000 animals in the late 1920s, then steadily rising harvest numbers to a peak of 80,000 to 100,00 animals in some years in the late 1950s, followed by a declining trend from the late 1960s onward (Longhurst et al., 1976; Webb, 2016). Moreover, annual estimates of the total deer population in California (*O. hemionus hemionus* and *O. hemionus columbianus* combined) were higher in the late 1950s (range from 1955 to 1959 of 1100,000–1200, 000 deer) than in the late 1990s (range from 1995 to 1999 of 531, 000–752,000 deer) and the late 2010s (range from 2015 to 2019 of 458, 000–637,000 deer) (Connolly, 1981; Jensen et al., 2023). A similar trend was documented from Oregon (Jensen et al., 2023). The declining trend in deer numbers from the 1960s onward in the far western US is in stark contrast to the dramatic increase in numbers seen for the white-tailed deer in the eastern US during the same time period (Adams and Hamilton, 2011).

We consider it likely that deer persisted through the late 1800s and early 1900s in sufficient numbers across the core geographic range of *I. pacificus* to allow the tick to maintain widely distributed populations, which then were documented along the Pacific Coast from southernmost California to northernmost Washington from the 1890s to the 1930s (see Sections 3.2-3.4). In California, *I. pacificus* adults were recovered from deer on several occasions already from 1903 to 1915 (Clarke 1912, 1913; CDPH and USNTC, unpublished data). Although deer populations then fluctuated from the 1920s until present (first increasing and then decreasing before more or less stabilizing in California and Oregon; Jensen et al., 2023), there is no clear evidence from the documented *I. pacificus* records that the tick has been limited in its geographic range over that time period. We speculate that deer have been abundant and widespread enough over the last 100 years to continuously support stable populations of *I. pacificus* across the tick’s present range in the far western US. This is in stark contrast to the northern part of the eastern US, where the decimation of white-tailed deer in the 1800s and early 1900s presumably led to the disappearance of *I. scapularis* from large parts of its previous range, and the subsequent reemergence of the white-tailed deer was followed by well documented spread and proliferation of *I. scapularis* across forested portions of the Upper Midwest and Northeast (Eisen and Eisen, 2023).

## 5. Association of a changing climate with the geographic distribution of *I. pacificus*

The impact a warming climate may have had on the geographic distribution and local abundance of *I. pacificus* in recent decades, and may have in the future, remains unclear. Modeling of abiotic and biotic factors associated with the current distribution of *I. pacificus* in California (Eisen et al., 2018) or across the full range of the tick (Hahn et al., 2016) indicate that tick presence is related to climate variables. Both models indicated that cold-season temperature and rainfall are important determinants of environmental suitability for *I. pacificus*, with warm and wet winters providing the most suitable climatic conditions. In the California model (Eisen et al., 2018), the likelihood of an area being classified as suitable for the tick was found to increase steadily with increasing temperatures >0 °C during the coldest quarter of the year, and further increased when precipitation ranged from 400 to 800 mm during the coldest quarter. The model based on the entire geographic range of *I. pacificus* (Hahn et al., 2016) provided similar results: the likelihood of an area being classified as suitable for the tick was found to increase with precipitation in the coldest and warmest quarters, and mean temperature of the wettest quarter (Hahn et al., 2016).

Not surprisingly, efforts to predict changes in the geographic distribution of *I. pacificus* under scenarios of climate warming indicate the tick may expand into higher elevations in mountainous areas of California, Oregon, and Washington, and northward along the Pacific Coast in British Columbia and into southernmost Alaska (Alkishe et al., 2021; Hahn et al., 2021; Witmer et al. 2022). The potential for range contraction in the warmest and driest portions of the current range, including parts of southern California, remains unclear. Future climate warming also may result in changes to the times of year when different life stages are questing for hosts, with variable impacts across climatically highly diverse states such as California (MacDonald et al., 2019, 2020b).

## 6. Future directions

Knowledge of the geographic distribution of *I. pacificus* accumulated via opportunistic collection from hosts and sporadic drag sampling efforts from the 1890s up to the early 1980s, when the incrimination of the tick as a vector of Lyme disease spirochetes resulted in the initiation of systematic drag sampling collections, particularly in California and Oregon, to clarify the range and local density of *I. pacificus* as well as infection with Lyme disease spirochetes. There are no clear indications that the geographic distribution and density of *I. pacificus* will change profoundly in the foreseeable future within the core portion of its range in California, western Oregon, and western Washington. However, future changes to precipitation and temperature patterns could impact both the distribution (expansion or contraction) and density (increase or decrease) of *I. pacificus*, particularly in settings where current environmental conditions are either marginally suitable or nearly suitable for the tick to survive and reproduce. Areas where the tick could potentially expand its range due to climate warming include higher elevations in the Sierra Nevada range, the Klamath Mountains, and the Cascade Range, as well as in coastal southernmost Alaska. Conversely, climate warming could potentially be detrimental to *I. pacificus* in southernmost California (including the mountain ranges on the South Coast and the South Interior zone) as well as inland locations in Arizona and Utah where the tick is present. Another potential scenario is that increased precipitation, should it occur, may allow the tick to establish populations in nearly suitable but currently too dry local environments in the Great Basin and Columbia Plateau zones in easternmost California, the eastern parts of Oregon and Washington, and western Utah. We encourage surveillance for *I. pacificus* based on schemes specifically designed to monitor climate-driven changes to the presence and density of the tick, from the present into the future, in core distributional areas as well as in those areas where climate change is expected to have the greatest impact on tick presence and density. As noted previously in the paper, it also would be interesting to re-sample some geographic outlier locations where *I. pacificus* was recorded in the past based on drag sampling or examination of local wildlife (for example the Hualapai Mountain County Park in northwestern Arizona and the Nevada Test Site in southern Nevada), and to conduct drag sampling in outlier locations where the evidence for presence of *I. pacificus* is based solely on recovery of ticks from humans or pets without knowledge of travel histories (for example Pend Oreille County in northeastern Washington and Bannock County in southeastern Idaho). We also encourage renewed studies of the evolutionary history and recent geographic spread patterns of *I. pacificus* using state-of-the-science genetic molecular approaches to better understand the potential for spread of this species within and beyond its current geographic range.

## 7. Disclaimer

The findings and conclusions of this study are by the authors and do not necessarily represent the views or opinions of the Centers for Disease Control and Prevention, the California Department of Public Health, or the California Health and Human Services Agency.

## Figures and Tables

**Table 1:** County classification for *Ixodes pacificus* as established or reported by state in the western United States by 1996 (Dennis et al., 1998), 2015 (Eisen et al., 2016a), and 2022 (CDC, 2023c).

State	Number of total counties in state	County status for *I. pacificus*^a^					
		1996 Established^b^	Reported^c^	2015 Established^b^	Reported^c^	2022 Established^b^	Reported^c^
California	58	55	1	55	1	55	1
Oregon	36	18	4	18	4	18	4
Washington	39	12	5	16	6	16	6
Utah	29	4	4	5	3	5	3
Nevada	16	0	2	0	2	0	2
Arizona	15	1	0	1	0	1	0
All states	193	90	16	95	16	95	16

a Counties not included in the sources for this table (Dennis et al., 1998; Eisen et al., 2016a; CDC, 2023c) but where *I. pacificus* may be present based on our review include Anchorage in Alaska (Hahn et al., 2020), Bannock in Idaho (Easton et al., 1977), Morrow and Yamhill in Oregon (Chamberlin, 1937; Doggett et al., 2008; Porter et al., 2021), and Grays Harbor and Pend Oreille in Washington (Porter et al., 2021). We encourage drag sampling efforts in these counties to provide definitive evidence of the presence of *I. pacificus*.

b Established: Six or more ticks or two or more tick life stages.

c Reported: Fewer than six ticks and one tick life stage only.

**Table 2:** References for county records of *Ixodes pacificus*, by state in the western United States.

State	County	References for county records of *Ixodes pacificus*^a^
Alaska	Anchorage^b^	Hahn et al. (2020)
Arizona	Mohave	Olson et al. (1992); Dennis et al. (1998); Eisen et al. (2016a)
California	Alameda	CDPH^c^; USNTC^d^; Cooley and Kohls (1945); Holdenried et al. (1951); Arthur and Snow (1968); Bissett and Hill (1987); Webb et al. (1990a); Schwan et al. (1993); Dennis et al. (1998); Hui et al. (1998, 2004); Kramer et al. (1999); Li et al. (2000); Fedorova et al. (2014); Padgett et al. (2014); Eshoo et al. (2015); Eisen et al. (2016a); Kirkpatrick et al. (2017); Kleinjan et al. (2019); Rose et al. (2019); Salkeld et al. (2019, 2021); Porter et al. (2021); James et al. (2022); Paddock et al. (2022)
California	Amador	CDPH; Schwan et al. (1993); Dennis et al. (1998); Hui et al. (2004); Padgett et al. (2014); Eisen et al. (2016a); Rose et al. (2019)
California	Butte	CDPH; Monsen et al. (1990, 1992); Webb et al. (1990a); Schwan et al. (1993); Dennis et al. (1998); Hui et al. (2004); Padgett et al. (2014); Eisen et al. (2016a); Rose et al. (2019); Salkeld et al. (2019); Porter et al. (2021)
California	Calaveras	CDPH; Brunetti (1965); Schwan et al. (1993); Dennis et al. (1998); Hui et al. (2004); Padgett et al. (2014); Eisen et al. (2016a); Rose et al. (2019); Salkeld et al. (2019); Porter et al. (2021)
California	Colusa	CDPH; Dennis et al. (1998); Hui et al. (2004); Padgett et al. (2014); Eisen et al. (2016a); Rose et al. (2019)
California	Contra Costa	CDPH; USNTC; Lane and Lavoie (1988); Lane and Pascocello (1989); Webb et al. (1990a); Kramer and Beesley (1993); Lane (1996); Peavey et al. (1997); Dennis et al. (1998); Kramer et al. (1999); Hui et al. (2004); Padgett and Bonilla (2011); Padgett et al. (2014); Salkeld et al. (2014b, 2019); Eisen et al. (2016a); Rose et al. (2019); Porter et al. (2021); Lilly et al. (2022); Paddock et al. (2022)
California	Del Norte	CDPH; USNTC; Kohls and Cooley (1937); Cooley and Kohls (1945); Arthur and Snow (1968); Bissett and Hill (1987); Schwan et al. (1993); Dennis et al. (1998); Hui et al. (2004); Foley and Piovia-Scott (2014); Padgett et al. (2014); Eshoo et al. (2015); Eisen et al. (2016a); Porter et al. (2021)
California	El Dorado	CDPH; USNTC; Brunetti (1965); Webb et al. (1990a); Schwan et al. (1993); Barlough et al. (1997); Dennis et al. (1998); Hui et al. (2004); Padgett et al. (2014); Eisen et al. (2016a); Rose et al. (2019); Salkeld et al. (2019); Hacker et al. (2021); Porter et al. (2021); Paddock et al. (2022)
California	Fresno	CDPH; USNTC; Brunetti (1965); Schwan et al. (1993); Dennis et al. (1998); Hui et al. (2004); Padgett et al. (2014); Eisen et al. (2016a); Salkeld et al. (2019); Osborne et al. (2020); Porter et al. (2021)
California	Glenn	CDPH; Schwan et al. (1993); Dennis et al. (1998); Hui et al. (2004); Padgett et al. (2014); Eshoo et al. (2015); Eisen et al. (2016a); Rose et al. (2019)
California	Humboldt	CDPH; USNTC; Banks (1908); Jellison (1934); Cooley and Kohls (1945); Arthur and Snow (1968); Botzler et al. (1980); Bissett and Hill (1987); Barlough et al. (1997); Dennis et al. (1998); Hui et al. (2004); Fritz et al. (2005); Foley et al. (2007, 2011); Gabriel et al. (2009); Nieto et al. (2009); Rejmanek et al. (2011); Foley and Piovia-Scott (2014); Padgett et al. (2014); Eshoo et al. (2015); Eisen et al. (2016a); Rose et al. (2019); Salkeld et al. (2019); Porter et al. (2021); Paddock et al. (2022)
California	Imperial	CDPH; Webb et al. (1990a); Dennis et al. (1998); Hui et al. (2004); Eisen et al. (2016a)
California	Inyo	CDPH; Dennis et al. (1998); Hui et al. (2004); Eisen et al. (2016a); Rose et al. (2019)
California	Kern	CDPH; USNTC; Webb et al. (1990a); Schwan et al. (1993); Dennis et al. (1998); Hui et al. (2004); Padgett et al. (2014); Eisen et al. (2016a); Rose et al. (2019)
California	Kings	CDPH; Dennis et al. (1998); Hui et al. (2004); Eisen et al. (2016a)
California	Lake	CDPH; USNTC; Bissett and Hill (1987); Dennis et al. (1998); Kramer et al. (1999); Wright et al. (2003b); Hui et al. (2004); Foley and Piovia-Scott (2014); Padgett et al. (2014); Eshoo et al. (2015); Eisen et al. (2016a); Ryan et al. (2016); Rose et al. (2019); Salkeld et al. (2019); Porter et al. (2021)
California	Lassen	CDPH; Dennis et al. (1998); Hui et al. (2004); Eisen et al. (2016a)
California	Los Angeles	CDPH; USNTC; Cooley and Kohls (1945); Arthur and Snow (1968); Bennett (1987); Webb et al. (1990a); Goldberg and Bursey (1991); Schwan et al. (1993); Barlough et al. (1997); Dennis et al. (1998); Hu et al. (2004a); Hui et al. (2004); Lane et al. (2013); Padgett et al. (2014); Eisen et al. (2016a); MacDonald and Briggs (2016); Rose et al. (2019); Salkeld et al. (2019); Porter et al. (2021)
California	Madera	CDPH; USNTC; Jellison (1934); Cooley and Kohls (1945); Arthur and Snow (1968); Schwan et al. (1993); Dennis et al. (1998); Hui et al. (2004); Padgett et al. (2014); Eisen et al. (2016a); Salkeld et al. (2019); Osborne et al. (2020)
California	Marin	CDPH; USNTC; Burgdorfer et al. (1985); Lane and Burgdorfer (1986, 1987); Lane and Lavoie (1988); Loye and Lane (1988); Lane and Pascocello (1989); Lane and Stubbs (1990); Webb et al. (1990a); Lane (1992); Dennis et al. (1998); Vredevoe et al. (1999); Li et al. (2000); Hui et al. (2004); Salkeld et al. (2008, 2014a, 2014b, 2015, 2019, 2021); Castro and Clover (2010); Foley et al. (2011); Rejmanek et al. (2011); Swei et al. (2011b, 2011c, 2012, 2015); Foley and Piovia-Scott (2014); Padgett et al. (2014); Eshoo et al. (2015); Eisen et al. (2016a); Nieto and Salkeld (2016); Rose et al. (2019); Porter et al. (2021); Lilly et al. (2022); Paddock et al. (2022)
California	Mariposa	CDPH; Furman and Loomis (1984); Schwan et al. (1993); Dennis et al. (1998); Kramer et al. (1999); Hui et al. (2004); Fleer et al. (2011); Foley et al. (2011); Padgett et al. (2014); Eisen et al. (2016a); Rose et al. (2019); Salkeld et al. (2019)
California	Mendocino	CDPH; USNTC; Cooley and Kohls (1945); Longhurst and Douglas (1953); Osebold et al. (1962); Browning and Lauppe (1964); Westrom (1975); Lane et al. (1981, 1992, 2005a, 2007, 2010); Philip et al. (1981); Burgdorfer et al. (1985); Westrom et al. (1985); Lane and Burgdorfer (1986, 1987, 1988); Bissett and Hill (1987); Lane and Lavoie (1988); Lane and Loye (1989, 1991); Lane and Pascocello (1989); Lane (1990a, 1990c); Lane and Stubbs (1990); Webb et al. (1990a); Brown and Lane (1992); Manweiler et al. (1992); Schwan et al. (1993); Clover and Lane (1995); Dennis et al. (1998); Tälleklint-Eisen and Eisen (1999); Tälleklint-Eisen and Lane (1999, 2000); Vredevoe et al. (1999); Eisen et al. (2001, 2002, 2003, 2004a, 2004b, 2004c, 2004d, 2006a, 2006b, 2016a); Slowik and Lane (2001); Casher et al. (2002); Hui et al. (2004); Nieto et al. (2009); Foley and Nieto (2011); Foley et al. (2011); Rejmanek et al. (2011); Foley and Piovia-Scott (2014); Padgett et al. (2014); Salkeld et al. (2014b, 2019, 2021); Eshoo et al. (2015); Rose et al. (2019); Porter et al. (2021)
California	Merced	CDPH; Dennis et al. (1998); Hui et al. (2004); Eisen et al. (2016a)
California	Mono	Dennis et al. (1998); Hui et al. (2004); Eisen et al. (2016a)
California	Monterey	CDPH; USNTC; Jellison (1934); Cooley and Kohls (1945); Linsdale and Tevis (1951); Linsdale and Tomich (1953); Arthur and Snow (1968); Philip et al. (1981); Bissett and Hill (1987); Webb et al. (1990a); Schwan et al. (1993); Dennis et al. (1998); Foley et al. (1999); Hui et al. (2004); Padgett et al. (2014); Eisen et al. (2016a); Rose et al. (2019); Salkeld et al. (2019, 2021); Porter et al. (2021); Paddock et al. (2022)
California	Napa	CDPH; Bissett and Hill (1987); Webb et al. (1990a); Dennis et al. (1998); Reubel et al. (1998); Hui et al. (2004); Wright et al. (2010, 2011, 2012); Phan et al. (2011); Dingler et al. (2014); Foley and Piovia-Scott (2014); Padgett et al. (2014); Salkeld et al. (2014a, 2015, 2019, 2021); Eshoo et al. (2015); Eisen et al. (2016a); Nieto and Salkeld (2016); Rose et al. (2019); Porter et al. (2021); Paddock et al. (2022)
California	Nevada	CDPH; Schwan et al. (1993); Dennis et al. (1998); Hui et al. (2004); Padgett et al. (2014); Eisen et al. (2016a); Rose et al. (2019); Salkeld et al. (2019); Porter et al. (2021); Paddock et al. (2022)
California	Orange	CDPH; USNTC; Cooley and Kohls (1945); Arthur and Snow (1968); Webb et al. (1990a, 1990b, 1992); Meyers et al. (1992); Schwan et al. (1993); Barlough et al. (1997); Dennis et al. (1998); Hui et al. (2004); Padgett et al. (2014); Eshoo et al. (2015); Eisen et al. (2016a); Billeter et al. (2017); Rose et al. (2019); Salkeld et al. (2019); Porter et al. (2021)
California	Placer	CDPH; USNTC; Bissett and Hill (1987); Schwan et al. (1993); Dennis et al. (1998); Reubel et al. (1998); Wright et al. (1998, 2000, 2003b); Vredevoe et al. (1999); Hui et al. (2004); Padgett et al. (2014); Eshoo et al. (2015); Eisen et al. (2016a); Rose et al. (2019); Salkeld et al. (2019); Porter et al. (2021); Paddock et al. (2022)
California	Plumas	CDPH; USNTC; Arthur and Snow (1968); Webb et al. (1990a); Dennis et al. (1998); Hui et al. (2004); Padgett et al. (2014); Eisen et al. (2016a); Porter et al. (2021)
California	Riverside	CDPH; USNTC; Cooley and Kohls (1945); Ryckman et al. (1955); Arthur and Snow (1968); Schwan et al. (1993); Dennis et al. (1998); Hu et al. (2004b); Hui et al. (2004); Padgett et al. (2014); Eisen et al. (2016a); Rose et al. (2019); Salkeld et al. (2019)
California	Sacramento	CDPH; Webb et al. (1990a); Wright (1992); Schwan et al. (1993); Dennis et al. (1998); Wright et al. (1996, 2003b); Hui et al. (2004); Padgett et al. (2014); Eisen et al. (2016a); Rose et al. (2019); Salkeld et al. (2019); Porter et al. (2021)
California	San Benito	CDPH; USNTC; Jellison (1934); Cooley and Kohls (1945); Osebold et al. (1962); Bissett and Hill (1987); Webb et al. (1990a); Schwan et al. (1993); Dennis et al. (1998); Hui et al. (2004); Padgett et al. (2014); Eisen et al. (2016a); Rose et al. (2019); Porter et al. (2021)
California	San Bernardino	CDPH; USNTC; Cooley and Kohls (1945); Ryckman et al. (1955); Boyce et al. (1992); Meyers et al. (1992); Schwan et al. (1993); Dennis et al. (1998); Hui et al. (2004); Padgett et al. (2014); Eshoo et al. (2015); Eisen et al. (2016a); Rose et al. (2019); Porter et al. (2021)
California	San Diego	CDPH; USNTC; Kohls and Cooley (1937); Webb et al. (1990a); Ryckman et al. (1955); Olson et al. (1993); Schwan et al. (1993); Dennis et al. (1998); Lang (1999); Hui et al. (2004); Padgett et al. (2014); Eisen et al. (2016a); Rose et al. (2019); Salkeld et al. (2019); Porter et al. (2021)
California	San Francisco	CDPH; USNTC; Cooley and Kohls (1945); Dennis et al. (1998); Hui et al. (2004); Eisen et al. (2016a); Salkeld et al. (2019); Porter et al. (2021)
California	San Joaquin	CDPH; Dennis et al. (1998); Hui et al. (2004); Padgett et al. (2014); Eisen et al. (2016a); Rose et al. (2019)
California	San Luis Obispo	CDPH; USNTC; Jellison (1934); Cooley and Kohls (1945); Webb et al. (1990a); Schwan et al. (1993); Dennis et al. (1998); Hui et al. (2004); Nieto et al. (2007); Lumbad et al. (2011); Padgett et al. (2014); Eisen et al. (2016a); Rose et al. (2019); Salkeld et al. (2019); Porter et al. (2021); Paddock et al. (2022)
California	San Mateo	CDPH; USNTC; Mohr et al. (1964); Webb et al. (1990a); Schwan et al. (1993); Dennis et al. (1998); Hui et al. (2004); Padgett et al. (2014); Salkeld et al. (2014a, 2014b, 2015, 2019, 2021); Eisen et al. (2016a); MacDonald et al. (2016); Nieto and Salkeld (2016); Rose et al. (2019); Crews and Roth (2020); Nakano et al. (2021); Porter et al. (2021); Lilly et al. (2022); Paddock et al. (2022)
California	Santa Barbara	CDPH; USNTC; Jellison (1934); Lane et al. (1982); Webb et al. (1990a); Schwan et al. (1993); Dennis et al. (1998); Crooks et al. (2001, 2004); Hui et al. (2004); Padgett et al. (2014); Eisen et al. (2016a); MacDonald and Briggs (2016); MacDonald et al. (2017, 2020a); MacDonald (2018); Rose et al. (2019); Salkeld et al. (2019); Porter et al. (2021); Paddock et al. (2022)
California	Santa Clara	CDPH; USNTC; Banks (1908); Nuttall et al. (1911); Jellison (1934); Cooley and Kohls (1945); Arthur and Snow (1968); Bissett and Hill (1987); Dennis et al. (1998); Chang et al. (2001); Hui et al. (2004); Padgett et al. (2014); Salkeld et al. (2014b, 2015, 2019, 2021); Bonilla (2016); Eisen et al. (2016a); Nieto and Salkeld (2016); Rose et al. (2019); Porter et al. (2021); Paddock et al. (2022)
California	Santa Cruz	CDPH; USNTC; Banks (1908); Webb et al. (1990a); Schwan et al. (1993); Barlough et al. (1997); Dennis et al. (1998); Kramer et al. (1999); Holden et al. (2003, 2006); Hui et al. (2004); Foley et al. (2011); Rejmanek et al. (2011); Foley and Piovia-Scott (2014); Padgett et al. (2014); Salkeld et al. (2014b, 2019, 2021); Eshoo et al. (2015); Eisen et al. (2016a); Nieto and Salkeld (2016); Rose et al. (2019); López-Pérez et al. (2021); Porter et al. (2021)
California	Shasta	CDPH; Kohls and Cooley (1937); Bissett and Hill (1987); Schwan et al. (1993); Barlough et al. (1997); Dennis et al. (1998); Hui et al. (2004); Padgett et al. (2014); Bonkrude (2016); Eisen et al. (2016a); Rose et al. (2019); Salkeld et al. (2019); Porter et al. (2021)
California	Sierra	CDPH; Dennis et al. (1998); Hui et al. (2004); Padgett et al. (2014); Eisen et al. (2016a); Rose et al. (2019)
California	Siskiyou	CDPH; USNTC; Schwan et al. (1993); Dennis et al. (1998); Hui et al. (2004); Padgett et al. (2014); Eisen et al. (2016a); Rose et al. (2019); Salkeld et al. (2019); Porter et al. (2021)
California	Solano	CDPH; USNTC; Schwan et al. (1993); Dennis et al. (1998); Hui et al. (2004); Padgett et al. (2014); Eisen et al. (2016a); Rose et al. (2019); Salkeld et al. (2019); Porter et al. (2021); Paddock et al. (2022)
California	Sonoma	CDPH; USNTC; Burgdorfer et al. (1985); Bissett and Hill (1987); Lane and Lavoie (1988); Lane and Pascocello (1989); Lane (1990b); Schwan et al. (1993); Barlough et al. (1997); Castro et al. (1997); Dennis et al. (1998); Kramer et al. (1999); Nicholson et al. (1999); Lane et al. (2001, 2006); Hui et al. (2004); Castro and Clover (2010); Swei et al. (2011a, 2015); Padgett et al. (2014); Salkeld et al. (2014a, 2015, 2019, 2021); Eshoo et al. (2015); Eisen et al. (2016a); Nieto and Salkeld (2016); Rose et al. (2019); Porter et al. (2021); Lilly et al. (2022); Paddock et al. (2022)
California	Stanislaus	CDPH; Dennis et al. (1998); Hui et al. (2004); Padgett et al. (2014); Eisen et al. (2016a); Rose et al. (2019)
California	Sutter	CDPH; Dennis et al. (1998); Wright et al. (2003a, 2003b, 2006); Hui et al. (2004); Foley et al. (2011); Eisen et al. (2016a)
California	Tehama	CDPH; Schwan et al. (1993); Dennis et al. (1998); Hui et al. (2004); Lane et al. (2005b); Padgett et al. (2014); Eisen et al. (2016a); Foley et al. (2016); Porter et al. (2021)
California	Trinity	CDPH; USNTC; Schwan et al. (1993); Dennis et al. (1998); Hui et al. (2004); Padgett et al. (2014); Eisen et al. (2016a); Rose et al. (2019); Salkeld et al. (2019); Porter et al. (2021)
California	Tulare	CDPH; Furman and Loomis (1984); Webb et al. (1990a); Schwan et al. (1993); Dennis et al. (1998); Hui et al. (2004); Padgett et al. (2014); Eisen et al. (2016a); Porter et al. (2021)
California	Tuolumne	CDPH; Schwan et al. (1993); Dennis et al. (1998); Hui et al. (2004); Padgett et al. (2014); Eisen et al. (2016a); Rose et al. (2019); Salkeld et al. (2019); Porter et al. (2021)
California	Ventura	CDPH; USNTC; Cooley and Kohls (1945); Nelson (1973); Webb et al. (1990a); Schwan et al. (1993); Dennis et al. (1998); Hui et al. (2004); Padgett et al. (2014); Eisen et al. (2016a); Rose et al. (2019); Salkeld et al. (2019); Porter et al. (2021)
California	Yolo	CDPH; Webb et al. (1990a); Schwan et al. (1993); Dennis et al. (1998); Wright et al. (1998, 2003b); Hui et al. (2004); Nieto and Foley (2009); Nieto et al. (2010); Dingler et al. (2014); Foley and Piovia-Scott (2014); Eisen et al. (2016a); Paddock et al. (2022)
California	Yuba	CDPH; Lane and Lavoie (1988); Lane and Pascocello (1989); Lane (1990c); Manweiler et al. (1990); Dennis et al. (1998); Hui et al. (2004); Padgett et al. (2014); Eisen et al. (2016a); Rose et al. (2019); Paddock et al. (2022)
Idaho	Bannock^b^	USNTC; Easton et al. (1977)
Nevada	Clark^b^	Beck et al. (1963)^e^; Dennis et al. (1998); Eisen et al. (2016a)
Nevada	Lincoln^b^	Beck et al. (1963)^e^; Dennis et al. (1998); Eisen et al. (2016a)
Oregon	Benton	USNTC; Chamberlin (1937); Kohls and Cooley (1937); Cooley and Kohls (1945); Easton and Goulding (1974); Easton et al. (1977); Dennis et al. (1998); Doggett et al. (2008); Eisen et al. (2016a)
Oregon	Clackamas	USNTC; Chamberlin (1937); Easton et al. (1977); Dennis et al. (1998); Doggett et al. (2008); Eisen et al. (2016a); Porter et al. (2021)
Oregon	Clatsop	USNTC; Easton et al. (1977); Dennis et al. (1998); Doggett et al. (2008); Eisen et al. (2016a)
Oregon	Columbia	Chamberlin (1937); Cooley and Kohls (1945); Easton et al. (1977); Dennis et al. (1998); Doggett et al. (2008); Eisen et al. (2016a); Porter et al. (2021)
Oregon	Coos	USNTC; Easton et al. (1977); Dennis et al. (1998); Doggett et al. (2008); Eisen et al. (2016a); Porter et al. (2021)
Oregon	Curry	USNTC; Chamberlin (1937); Cooley and Kohls (1945); Arthur and Snow (1968); Easton et al. (1977); Dennis et al. (1998); Doggett et al. (2008); Eisen et al. (2016a); Porter et al. (2021)
Oregon	Douglas	USNTC; Chamberlin (1937); Kohls and Cooley (1937); Arthur and Snow (1968); Hughes et al. (1976); Easton et al. (1977); Burgdorfer et al. (1985); Dennis et al. (1998); Doggett et al. (2008); Eisen et al. (2016a); Porter et al. (2021)
Oregon	Hood River	USNTC; Arthur and Snow (1968); Easton et al. (1977); Dennis et al. (1998); Doggett et al. (2008); Eisen et al. (2016a); Porter et al. (2021)
Oregon	Jackson	USNTC; Chamberlin (1937); Hughes et al. (1976); Easton et al. (1977); Dennis et al. (1998); Burkot et al. (1999); Doggett et al. (2008); Eisen et al. (2016a); Porter et al. (2021)
Oregon	Jefferson	Dennis et al. (1998); Eisen et al. (2016a)
Oregon	Josephine	USNTC; Chamberlin (1937); Davis and Kohls (1937); Kohls and Cooley (1937); Cooley and Kohls (1945); Arthur and Snow (1968); Hughes et al. (1976); Easton et al. (1977); Burgdorfer et al. (1985); Dennis et al. (1998); Burkot et al. (1999); Doggett et al. (2008); Eisen et al. (2016a); Porter et al. (2021)
Oregon	Lane	USNTC; Cooley and Kohls (1945); Easton and Goulding (1974); Easton et al. (1977); Dennis et al. (1998); Doggett et al. (2008); Eisen et al. (2016a); Porter et al. (2021)
Oregon	Lincoln	USNTC; Chamberlin (1937); Easton et al. (1977); Dennis et al. (1998); Doggett et al. (2008); Eisen et al. (2016a); Porter et al. (2021)
Oregon	Linn	USNTC; Easton et al. (1977); Dennis et al. (1998); Doggett et al. (2008); Eisen et al. (2016a); Porter et al. (2021)
Oregon	Marion	USNTC; Easton et al. (1977); Dennis et al. (1998); Doggett et al. (2008); Eisen et al. (2016a); Porter et al. (2021)
Oregon	Morrow^b^	Chamberlin (1937)
Oregon	Multnomah	USNTC; Chamberlin (1937); Hughes et al. (1976); Easton et al. (1977); Dennis et al. (1998); Doggett et al. (2008); Eisen et al. (2016a)
Oregon	Polk	Easton et al. (1977); Dennis et al. (1998); Doggett et al. (2008); Eisen et al. (2016a); Porter et al. (2021)
Oregon	Sherman	USNTC; Easton et al. (1977); Dennis et al. (1998); Doggett et al. (2008); Eisen et al. (2016a)
Oregon	Tillamook	USNTC; Hughes et al. (1976); Easton et al. (1977); Dennis et al. (1998); Doggett et al. (2008); Eisen et al. (2016a)
Oregon	Umatilla	USNTC; Cooley and Kohls (1945); Dennis et al. (1998); Eisen et al. (2016a)
Oregon	Wasco	USNTC; Easton et al. (1977); Dennis et al. (1998); Doggett et al. (2008); Eisen et al. (2016a); Porter et al. (2021)
Oregon	Washington	USNTC; Easton et al. (1977); Dennis et al. (1998); Doggett et al. (2008); Eisen et al. (2016a); Porter et al. (2021)
Oregon	Yamhill^b^	Doggett et al. (2008); Porter et al. (2021)
Utah	Beaver	Bishopp and Trembley (1945); Dennis et al. (1998); Eisen et al. (2016a)
Utah	Juab	USNTC; Beck (1955); Marchette et al. (1962); Dennis et al. (1998); Eisen et al. (2016a)
Utah	Millard	Edmunds (1951); Dennis et al. (1998); Davis et al. (2015); Eisen et al. (2016a)
Utah	Piute	USNTC; Bishopp and Trembley (1945); Dennis et al. (1998); Eisen et al. (2016a)
Utah	Salt Lake	Dennis et al. (1998); Eisen et al. (2016a)
Utah	Tooele	USNTC; Allred et al. (1960); Johnson (1966); Dennis et al. (1998); Davis et al. (2015); Eisen et al. (2016a)
Utah	Utah	USNTC; Beck (1955); Allred et al. (1960); Dennis et al. (1998); Eisen et al. (2016a)
Utah	Washington	Allred et al. (1960); Dennis et al. (1998); Davis et al. (2015); Eisen et al. (2016a)
Washington	Chelan	Dennis et al. (1998); Eisen et al. (2016a); Porter et al. (2021)
Washington	Clallam	Eisen et al. (2016a); Dykstra et al. (2020); Porter et al. (2021)
Washington	Clark	USNTC; Dennis et al. (1998); Eisen et al. (2016a)
Washington	Cowlitz	Dennis et al. (1998); Eisen et al. (2016a)
Washington	Grays Harbor^b^	Porter et al. (2021)
Washington	Island	Dennis et al. (1998); Eisen et al. (2016a)
Washington	Jefferson	Dennis et al. (1998); Eisen et al. (2016a); Dykstra et al. (2020)
Washington	King	Arthur and Snow (1968); Dennis et al. (1998); Eisen et al. (2016a); Porter et al. (2021)
Washington	Kitsap	Dennis et al. (1998); Eisen et al. (2016a); Porter et al. (2021)
Washington	Kittitas	Eisen et al. (2016a)
Washington	Klickitat	USNTC; Dennis et al. (1998); Eisen et al. (2016a); Dykstra et al. (2020); Porter et al. (2021)
Washington	Lewis	USNTC; Dennis et al. (1998); Eisen et al. (2016a); Porter et al. (2021)
Washington	Mason	USNTC; Dennis et al. (1998); Eisen et al. (2016a); Dykstra et al. (2020); Porter et al. (2021)
Washington	Okanogan	Eisen et al. (2016a)
Washington	Pacific	Eisen et al. (2016a)
Washington	Pend Oreille^b^	Porter et al. (2021)
Washington	Pierce	USNTC; Cooley and Kohls (1945); Dennis et al. (1998); Eisen et al. (2016a); Dykstra et al. (2020)
Washington	San Juan	USNTC; Kohls and Cooley (1937); Cooley and Kohls (1945); Dennis et al. (1998); Eisen et al. (2016a)
Washington	Skagit	USNTC; Dennis et al. (1998); Eisen et al. (2016a)
Washington	Skamania	Dennis et al. (1998); Eisen et al. (2016a); Porter et al. (2021)
Washington	Snohomish	Dennis et al. (1998); Eisen et al. (2016a)
Washington	Thurston	USNTC; Dennis et al. (1998); Eisen et al. (2016a); Dykstra et al. (2020); Porter et al. (2021)
Washington	Whatcom	USNTC; Arthur and Snow (1968); Dennis et al. (1998); Eisen et al. (2016a)
Washington	Yakima	Eisen et al. (2016a); Dykstra et al. (2020); Porter et al. (2021)

a Excluding records from Arthur and Snow (1968), Furman and Loomis (1984), and MacDonald et al. (2019, 2020b) represented only as point locations on maps without county boundaries.

b Drag sampling efforts in this county are encouraged to provide definitive evidence of the presence of *I. pacificus*.

c CDPH: California Department of Public Health, unpublished data.

d USNTC: United States National Tick Collection, unpublished data.

e Reported as the Nevada Test Site, not by county.

## Data Availability

No data was used for the research described in the article.
